# Circulating Homocysteine and Choroid Plexus Volume Across the Alzheimer’s Disease Continuum: Cross-Sectional and Progression-Related Associations

**DOI:** 10.3390/biology15161423

**Published:** 2026-08-18

**Authors:** Chenjie Feng, Tian Zhang, Xianglong Liu, Zhe Liu, Yu Zhao, Peng Zhang

**Affiliations:** 1 College of Medical Information and Engineering, Ningxia Medical University, Yinchuan 750004, China; chenjief@nxmu.edu.cn (C.F.); zhangtian@nxmu.edu.cn (T.Z.); liuzhe@nxmu.edu.cn (Z.L.); 2 School of Public Health, Ningxia Medical University, Yinchuan 750004, China; 18195136912@163.com; 3 Ningxia Key Laboratory of Environmental Factors and Chronic Disease Control, Yinchuan 750004, China

**Keywords:** Alzheimer’s disease, homocysteine, choroid plexus volume, mild cognitive impairment, single-nucleus RNA sequencing, spatial transcriptomics

## Abstract

Background: Elevated plasma homocysteine (HCY) is a risk factor for Alzheimer’s disease (AD), but its relationship with structural brain changes across the AD continuum remains unclear. The choroid plexus (CP) regulates cerebrospinal fluid homeostasis and may interface with peripheral metabolic signals. Whether HCY relates to CP structural alterations and disease progression remains unknown. Methods: We analyzed 819 Alzheimer’s Disease Neuroimaging Initiative (ADNI) participants (229 cognitively normal (CN), 397 with mild cognitive impairment (MCI), and 193 with AD dementia). Multinomial logistic regression assessed associations between HCY and diagnosis under stepwise covariate adjustment. Phenotype-wide structural magnetic resonance imaging (MRI) mapping identified HCY-associated signals. Cox models evaluated associations of CP volume (CPV) with CN-to-MCI and MCI-to-AD dementia conversion and whether CPV added prognostic discrimination beyond baseline disease-severity markers. Independent human CP single-nucleus and spatial transcriptomic datasets were reanalyzed to characterize epithelial expression states and their spatial organization in a hypothesis-generating analysis. Results: Higher HCY was associated with MCI and AD dementia; however, the AD association attenuated after adjustment for renal function, vitamin B12, and medications, whereas the MCI association remained stable. CPV was among the HCY-associated MRI signals that persisted after progressive covariate adjustment. Right and bilateral CPV showed model-dependent associations with MCI-to-AD dementia conversion. In the disease-severity sensitivity analysis, larger right and bilateral CPV remained associated with a higher risk of progression from MCI to AD dementia. Single-nucleus analysis identified two CP epithelial states with relatively high expression of one-carbon metabolism-related genes, termed one-carbon metabolism-enriched epithelial state A (OCM-Epi-A) and state B (OCM-Epi-B). Donor-level pseudobulk analysis did not identify pathway enrichment after false discovery rate correction, whereas OCM-Epi-A–like spots were located near endothelial spots more often than expected by chance in three of the four spatial samples. Conclusions: Circulating HCY was associated with larger CPV, and larger CPV showed model-dependent associations with MCI-to-AD dementia progression. Independent transcriptomic reanalysis identified one-carbon metabolism-enriched epithelial states and their spatial organization in postmortem CP tissue, providing hypothesis-generating tissue-level context for the ADNI associations.

## Simple Summary

Alzheimer’s disease progression involves complex changes in both the brain and the body, but the biological links between peripheral metabolic alterations and brain structural changes remain unclear. This study examined whether elevated blood homocysteine levels were associated with larger choroid plexus volume, an MRI-derived measure of the choroid plexus, a structure involved in maintaining the brain environment. In a large cohort covering normal cognition, mild cognitive impairment, and Alzheimer’s disease dementia, higher blood homocysteine levels were associated with increased choroid plexus volume, and larger choroid plexus volume was associated with a higher risk of progression from mild cognitive impairment to Alzheimer’s disease dementia. Exploratory reanalysis of independent postmortem single-nucleus and spatial transcriptomic datasets identified choroid plexus epithelial expression states involving one-carbon metabolism-related genes and spatial organization near vascular compartments. These independent transcriptomic findings provide tissue-level biological context for the associations observed in the Alzheimer’s Disease Neuroimaging Initiative (ADNI) and support further validation of these epithelial expression states.

## 1. Introduction

Alzheimer’s disease (AD) is increasingly recognized as a continuous biological and clinical process rather than a disorder that begins only at the dementia stage [1]. Pathological changes may occur years before overt functional impairment, and clinical symptoms often evolve gradually from cognitively normal status to mild cognitive impairment (MCI) and then to AD dementia. Within this continuum, MCI represents a particularly important stage because patients already show measurable cognitive decline while daily function remains relatively preserved [2]. However, MCI is highly heterogeneous: some individuals progress to AD dementia, whereas others remain stable or even return to normal cognition. Current biomarker-based frameworks emphasize that AD-related progression should not be interpreted solely on the basis of clinical diagnosis, but should be understood by integrating molecular, imaging, and clinical information [3,4]. Therefore, identifying biological signals associated with cognitive worsening and disease progression at early stages of the AD continuum remains important for understanding disease heterogeneity and improving risk stratification.

Homocysteine (HCY) is a sulfur-containing amino acid involved in methionine cycling and one-carbon metabolism [5]. It can be measured in peripheral blood, and its level is influenced by folate, vitamin B12, vitamin B6, renal function, and vascular-metabolic status, making it a clinically relevant marker for investigation. Previous epidemiological studies and meta-analyses have reported that higher plasma HCY levels are associated with increased risks of cognitive decline, dementia, and AD [6,7]. Neuroimaging studies also suggest that HCY may be related to brain structural changes. In studies using data from the Alzheimer’s Disease Neuroimaging Initiative (ADNI), higher HCY levels were associated with regional brain atrophy and cortical gray matter thinning involving frontal, parietal, occipital, and temporal regions [8,9]. In addition, B-vitamin treatment that lowers HCY has been reported to slow the rate of brain atrophy in older adults with MCI [10], whereas a similar intervention in patients with mild-to-moderate AD did not show clear cognitive benefit [11]. These findings suggest that the relevance of HCY may be more closely related to early structural vulnerability and progression risk than to its use as a single therapeutic target in established dementia.

Structural magnetic resonance imaging (MRI) is an important tool for assessing neurodegenerative injury across the AD continuum. Previous studies have mainly focused on classical structural regions, including the hippocampus, medial temporal lobe, entorhinal cortex, temporal cortex, cingulate structures, and temporoparietal cortex [12]. Atrophy or cortical thinning in these regions has been associated with memory impairment, cognitive decline, and conversion from MCI to AD dementia [13,14,15]. However, AD-related structural changes are not limited to neuronal-rich cortical and limbic regions. In recent years, choroid plexus volume (CPV) has received increasing attention as an emerging MRI phenotype. The choroid plexus (CP) is located within the ventricular system and plays important roles in cerebrospinal fluid production, blood–cerebrospinal fluid barrier function, immune surveillance, and maintenance of the brain fluid environment [16,17]. Recent MRI studies have shown that larger CPV is associated with the severity of cognitive impairment across the AD clinical spectrum, amyloid burden, poorer cognitive performance, and markers related to white matter microstructure, glymphatic function, and peripheral inflammation [18,19,20]. CPV has also been proposed as a candidate imaging marker in the AD continuum and has been associated with cognitive function in older adults without dementia [21,22]. Nevertheless, previous studies have mainly emphasized associations between CPV and cognitive impairment or AD-related pathological features. Whether CPV provides progression-related information beyond traditional structural MRI markers remains to be clarified. Notably, the CP is also an important interface through which peripheral metabolic status may influence the cerebrospinal fluid environment. Previous studies have shown that the CP is involved in the transport of folate and other one-carbon metabolism-related molecules into cerebrospinal fluid and brain tissue [23]. In patients with AD, cerebrospinal fluid folate levels are reduced, and cerebrospinal fluid HCY levels are negatively correlated with folate levels [24]. These findings suggest a potential link between one-carbon metabolism abnormalities and CP-related cerebrospinal fluid environmental changes. However, whether plasma HCY, as a peripheral marker related to one-carbon metabolism, is also associated with macroscopic structural changes of the CP remains insufficiently studied.

On this basis, CPV as an MRI-derived measure still has important limitations. MRI can show whether the CP is enlarged, but it cannot determine which cell types or tissue processes underlie this change. The CP is composed of epithelial cells, vascular-associated cells, stromal cells, and immune cells, and its volume change may reflect different processes, such as altered barrier function, vascular responses, immune activation, matrix deposition, or tissue remodeling. Therefore, cell-type-resolved transcriptomic data may be used to characterize candidate CP cellular states and tissue-level expression patterns that provide independent biological context for the imaging associations. Single-cell and single-nucleus transcriptomic studies have shown that cell-type-resolved analyses can reveal disease-related cellular states that are difficult to detect using bulk tissue analysis or imaging measures alone [25]. Recent single-cell work in the CP of AD models further supports the value of examining abnormalities in this tissue at the cellular level [26]. Thus, reanalysis of independent CP single-cell and spatial transcriptomic data may help characterize epithelial transcriptional states and their spatial organization, providing hypothesis-generating biological context for the imaging associations.

In this study, we used multimodal data from the Alzheimer’s Disease Neuroimaging Initiative (ADNI) to systematically evaluate the relationships among circulating HCY, cognitive impairment, and CPV across the AD continuum. First, we examined whether HCY was associated with AD- and MCI-related clinical status. We then tested whether HCY was associated with CPV after adjustment for relevant demographic and clinical factors. Next, we assessed whether CPV provided stage-specific information on disease conversion, with particular attention to progression from MCI to AD dementia. Finally, we reanalyzed independent CP single-nucleus and spatial transcriptomic datasets to characterize one-carbon metabolism-related epithelial expression patterns and their spatial context. Because these datasets lacked donor-specific HCY measurements and sample-level diagnostic linkage, this component was intended to generate tissue-level hypotheses rather than validate an HCY-mediated mechanism. This study aimed to clarify whether HCY is associated with a CP-related structural phenotype and whether CPV may help characterize progression risk within the AD continuum.

## 2. Methods

### 2.1. Study Design and Analytic Cohorts

Data used in this study were obtained from the Alzheimer’s Disease Neuroimaging Initiative (ADNI) database (https://adni.loni.usc.edu/, accessed on 2 March 2026), a public–private partnership launched in 2003 to track the progression of mild cognitive impairment and early AD using combined clinical, imaging, and biological markers. Participants were included in the diagnostic and structural magnetic resonance imaging (MRI) analyses according to the availability of valid homocysteine (HCY) measurements and the data required for each analysis. For each participant, the HCY index visit was defined as the first visit with a valid HCY measurement that passed the study quality-control criteria. The corresponding ADNI REGISTRY visit date was used as the index HCY date. For the structural MRI analyses, the index HCY measurement was paired with the first eligible structural MRI examination acquired on or after the index HCY date, based on the actual MRI acquisition date. Baseline diagnosis was classified as cognitively normal (CN), MCI, or AD dementia according to ADNI diagnosis fields. Participants were excluded from baseline diagnostic analyses if they had invalid or non-positive HCY values, or if a valid baseline diagnosis was not available.

For the structural MRI analyses, temporal eligibility was evaluated using the dates of the HCY measurement and MRI acquisition. The index exposure was the first quality-controlled HCY measurement that could be paired with an MRI examination acquired on or after the HCY date. Participants without a temporally eligible MRI examination were excluded from the MRI cohort. These criteria yielded a time-ordered MRI cohort of 749 participants. After excluding 40 participants with incomplete C1–C4 covariate data, 709 participants constituted the fixed common complete-case sample used in the primary structural MRI analyses. Baseline diagnosis, the index HCY measurement, the paired MRI examination, and subsequent conversion or censoring events were temporally aligned, with all diagnostic conversion events included in the prospective analyses occurring after the paired MRI examination.

Longitudinal diagnosis records were used to define two progression cohorts anchored to the index MRI examination. For each participant, the first quality-controlled HCY measurement was paired with the earliest eligible structural MRI obtained on or after that measurement, and this examination was designated the index MRI. Only HCY measurements obtained on or before the index MRI date were used; post-MRI and post-conversion HCY measurements were not included in the primary imaging analyses. Baseline diagnostic status was assigned from the diagnosis associated with the index MRI visit. The CN-to-MCI cohort included participants who were cognitively normal at the index MRI and were followed from that examination until the first subsequent MCI diagnosis or the last available diagnostic assessment. For the CN-to-MCI analysis, participants who developed dementia without a recorded intervening MCI diagnosis were not counted as CN-to-MCI events; instead, their follow-up was censored on the date of dementia diagnosis. The MCI-to-AD dementia cohort included participants who had MCI at the index MRI and were followed until the first subsequent AD dementia diagnosis or the last available diagnostic assessment.

### 2.2. HCY Exposure Definitions

HCY concentrations (μmol/L) were analyzed after invalid or non-positive measurements were excluded. HCY values were log-transformed and standardized so that effect estimates corresponded to a one-standard-deviation (SD) increase. The primary exposure for the structural MRI analyses was the standardized log-HCY value measured at the HCY index visit. In supplementary sensitivity analyses, participants with at least two valid HCY measurements obtained on distinct dates strictly before the paired MRI examination were evaluated using the mean, maximum, and time-normalized area under the curve (AUC) of pre-MRI log-HCY. The AUC was calculated by trapezoidal integration and divided by the elapsed time between the first and last included measurements. Each alternative HCY summary was standardized within the repeated-measurement subset. HCY measurements obtained after MRI acquisition were excluded from all exposure summaries.

### 2.3. Stepwise Covariate Adjustment

Adjusted analyses used the prespecified C1–C4 covariate framework. C1 included standard clinical covariates; C2 further incorporated vascular and comorbidity variables; C3 additionally included laboratory measurements; and C4 further included medication indicators, as detailed in Table 1. For the prospective diagnostic-conversion analyses based on index CPV, C1–C4 were fitted within a fixed outcome-specific cohort so that all models for a given progression outcome used the same participants, event indicators, and follow-up times. Missing adjustment covariates were addressed using multiple imputation as described in Section 2.6.

An additional CPV-specific neuroimaging sensitivity model, designated C1-N, was evaluated separately from the sequential C1–C4 framework. C1-N extended C1 by additionally adjusting for scanner batch, MRI field strength, laterality-matched lateral ventricular volume, total cerebrospinal fluid volume, total gray matter volume, and laterality-matched hippocampal volume. Left-sided models used left ventricular and hippocampal volumes, right-sided models used the corresponding right-sided measures, and bilateral models used the sums of the left and right measurements obtained from the same index MRI. Acquisition site had already been addressed in the consortium ComBat harmonization and was not entered as a separate study-level model term. C1-N was designed to evaluate acquisition-related and structural-anatomical confounding of CPV; white matter hypointensity burden was instead included in the separate S1 progression sensitivity analysis evaluating prognostic associations beyond baseline disease severity. C1-N was treated as a separate neuroimaging sensitivity model rather than as a fifth sequential covariate tier.

### 2.4. Diagnostic-Stage Analyses

Baseline HCY distributions across the CN, MCI, and AD dementia groups were compared using the Kruskal–Wallis test, followed by pairwise two-sided Mann–Whitney U tests with Benjamini–Hochberg (BH) false discovery rate (FDR) correction. Associations between the log-HCY z-score and diagnostic stage were evaluated via multinomial logistic regression, designating the CN group as the reference. Exponentiated model coefficients were reported as relative risk ratios (RRRs) with 95% confidence intervals (CIs). Primary diagnostic-stage visualizations depict outcomes from the C1 through C4 models. To quantify the impact of comprehensive covariate adjustment on the HCY association, the attenuation from C1 to C4 was calculated on the log-RRR scale. Using the fully adjusted C4 model, we estimated the adjusted probabilities of being in the CN, MCI, or AD dementia stage across different HCY levels; during prediction, the other covariates were kept at their observed distributions in the study cohort. Density and bar plots were used only for sample characterization.

### 2.5. Association of Index HCY with Prespecified Structural MRI Phenotypes

To evaluate the structural MRI correlates of index HCY, standardized log-transformed index HCY was examined in relation to 42 prespecified structural MRI phenotypes encompassing cortical thickness, regional brain volumes, ventricular and cerebrospinal fluid volumes, white matter hypointensities, and choroid plexus volume. Continuous MRI phenotypes were standardized before analysis and modeled as outcomes using ordinary least-squares regression with type 3 heteroscedasticity-consistent (HC3) standard errors. For volumetric phenotypes, intracranial volume was included as an additional covariate, except for measures that had already been normalized or were expressed as ratios. The coefficient, robust standard error, 95% confidence interval, and *p* value for the index HCY term were recorded.

C1 served as the primary screening model. The *p* values for the index HCY term were jointly adjusted across all 42 MRI phenotypes using the Benjamini–Hochberg procedure, with FDR < 0.05 defining statistical significance. Phenotypes meeting this threshold in C1 were subsequently evaluated in the progressively adjusted C2–C4 sensitivity models. C1–C4 were fitted using the same fixed common complete-case sample to limit changes in effect estimates attributable to differences in sample composition. Because the phenotypes evaluated in C2–C4 were selected on the basis of the C1 screening results, the corresponding C2–C4 *p* values were interpreted as nominal sensitivity results and were not subjected to separate FDR correction.

The associations of index HCY with left, right, and bilateral choroid plexus volume were additionally evaluated using the CPV-specific C1-N model defined in Section 2.3. C1-N was analyzed in a separate complete-case neuroimaging sample determined by the availability of its additional imaging covariates and was not required to have the same sample size as the fixed C1–C4 analysis sample. For each CPV laterality, C1 was refitted in the corresponding C1-N sample to distinguish changes associated with the additional neuroimaging adjustment from those attributable to sample composition. The *p* values from the left, right, and bilateral C1-N analyses were jointly adjusted using the Benjamini–Hochberg procedure as a separate family of three sensitivity tests. The same-sample C1 refits were used to compare effect estimates and did not replace the original C1 screening results or redefine its 42-phenotype FDR family.

In supplementary repeated-measurement sensitivity analyses, the index HCY exposure was replaced with the mean, maximum, or time-normalized area under the curve of eligible pre-MRI log-transformed HCY measurements among participants with at least two such measurements. Each alternative exposure summary was standardized and evaluated using the same C1 42-phenotype regression framework. Benjamini–Hochberg correction was applied across all 42 phenotypes within each alternative HCY definition, with each definition treated as a separate testing family.

### 2.6. CP Volume Processing and Survival Analyses

Structural MRI data were obtained from high-resolution three-dimensional T1-weighted ADNI acquisitions using magnetization-prepared rapid gradient-echo (MPRAGE) or vendor-equivalent inversion recovery–prepared spoiled gradient-recalled echo (IR-SPGR) protocols (https://adni.loni.usc.edu/data-samples/adni-data/neuroimaging/mri/, accessed on 2 March 2026). In the 709-participant fixed C1–C4 complete-case MRI sample, 553 scans (78.0%) were acquired at 1.5 T, 30 (4.2%) at 2.9 T, and 126 (17.8%) at 3.0 T. Scanner manufacturers were Siemens (*n* = 340), GE Medical Systems (*n* = 254), and Philips Medical Systems (*n* = 115). Scan-specific acquisition dates, field strengths, manufacturers, and scanner-batch variables were obtained from the Alzheimer’s Disease Sequencing Project (ADSP) Phenotype Harmonization Consortium (PHC) phenotype table.

Choroid plexus volumes were obtained from the ADSP PHC T1 FreeSurfer release. The ADSP PHC processing methods specify that these measures were generated using a fully automated cross-sectional FreeSurfer version 6.0 pipeline with standard recon-all processing (https://adni.loni.usc.edu/data-samples/adni-data/neuroimaging/mri/mri-numerical-data-sets/, accessed on 2 March 2026); the general FreeSurfer processing framework has been described previously [27]. The released left and right CPV measures correspond to the standard FreeSurfer subcortical labels Left-choroid-plexus (label 31) and Right-choroid-plexus (label 63) (https://surfer.nmr.mgh.harvard.edu/fswiki/FsTutorial/AnatomicalROI/FreeSurferColorLUT, accessed on 2 March 2026). Bilateral CPV was calculated as the same-scan sum of the released left and right values. The statistical analyses used the PHC-released scalar measures without reprocessing the analytic cohort; FreeSurfer was rerun only for selected illustrative scans. For the progression analyses, each CPV measure was regressed on intracranial volume (ICV), and the resulting residuals were standardized to generate left, right, and bilateral ICV-residualized CPV z-scores; hazard ratios were interpreted per one-SD higher ICV-residualized CPV.

The PHC processing documentation reports visual examination of FreeSurfer surface parcellations following the Enhancing NeuroImaging Genetics through Meta-Analysis (ENIGMA) quality-control protocol. Outputs were classified as failure, lower-quality pass, or full pass; ratings 2 and 3 were retained, and cases classified as uncertain between failure and a lower-quality pass were adjudicated by a second reviewer (https://adni.loni.usc.edu/data-samples/adni-data/neuroimaging/mri/mri-numerical-data-sets/, accessed on 2 March 2026). ADNI acquisition-level quality control additionally included automated protocol checks and trained manual inspection for artifacts, anatomical coverage, slice completeness, and overall image quality (https://adni.loni.usc.edu/data-samples/adni-data/neuroimaging/mri/mri-quality-control/, accessed on 2 March 2026). The released PHC table contained 10,260 processed observations from 2504 participants, with nonmissing left CPV, right CPV, and intracranial volume in all released rows. This table-level availability was not interpreted as a segmentation-failure rate because unsuccessful or unreleased processing attempts and per-scan quality-control (QC) logs were not included in the released phenotype table. No additional winsorization, interquartile-range-based exclusion, or statistical outlier removal was applied to CPV. Standardization and intracranial-volume residualization were analytic transformations rather than outlier-removal procedures.

To visualize the anatomical labels underlying the CPV measures, one T1-weighted scan from each diagnostic category (CN, MCI, and AD dementia) was processed separately using the FreeSurfer version 6.0 cross-sectional recon-all workflow [27]. Left-choroid-plexus (label 31) and Right-choroid-plexus (label 63) masks were extracted from the resulting aseg.mgz files and overlaid on the corresponding T1-weighted images (https://surfer.nmr.mgh.harvard.edu/fswiki/FsTutorial/AnatomicalROI/FreeSurferColorLUT, accessed on 3 March 2026). For each scan, the coronal and axial sections containing the largest combined number of label-31 and label-63 voxels were selected for display. Processing completion and nonempty label masks were verified from output logs and voxel counts, and the overlays were visually reviewed for anatomical localization. These representative images were prepared solely to illustrate label localization; all quantitative analyses used the PHC-released scalar CPV measures.

The PHC values had been harmonized by the consortium using Longitudinal ComBat to account for scanner platform and model, acquisition site, and field strength while preserving age, sex, race and ethnicity, and diagnosis as biological variables [28,29,30,31]. This study did not refit ComBat or modify the consortium harmonization model. Scanner batch and field strength were retained as acquisition variables in the C1-N sensitivity analysis.

To evaluate whether CPV was associated with progression beyond conventional indicators of baseline Alzheimer’s disease severity, we performed a post hoc disease-severity sensitivity analysis (S1) in the fixed MCI-to-AD dementia cohort. S1 extended the C1 covariates by adding baseline Clinical Dementia Rating–Sum of Boxes (CDR-SB), scanner batch, laterality-matched hippocampal and entorhinal measures, and white matter hypointensity burden; an alternative sensitivity model replaced CDR-SB with the Mini-Mental State Examination (MMSE). Missing non-outcome S1 covariates were imputed using predictive mean matching in 50 chained-equation datasets. CPV, event status, follow-up time, index MRI date, and MRI-derived measures were not imputed. A separate exploratory molecular-biomarker sensitivity analysis (S2) additionally adjusted for observed pre-index cerebrospinal fluid (CSF) amyloid-beta 42 and phosphorylated tau and was restricted to the 62 participants with both molecular biomarkers available; the molecular biomarkers were not imputed. Incremental prognostic contribution was evaluated using multiply imputed nested-model tests, Harrell’s C-index, and inverse-probability-of-censoring-weighted 3- and 5-year time-dependent AUCs. Apparent and optimism-corrected changes in discrimination were estimated using 500 paired participant-level bootstrap replicates.

Cox proportional hazards models were used to examine the associations of left, right, and bilateral CPV with CN-to-MCI and MCI-to-AD dementia progression. Separate fixed analysis cohorts were constructed for the two outcomes. Eligibility required complete CPV measurements, valid index dates, established temporal ordering between the index HCY measurement and index MRI, and complete event indicators and follow-up times. CPV, event status, follow-up time, index dates, and HCY-MRI temporal ordering were not imputed. Missing adjustment covariates were addressed separately within each progression cohort using multiple imputation by chained equations. The event indicator and Nelson-Aalen estimate of the cumulative baseline hazard were included in the imputation model. The same 50 imputed datasets were used to fit C1, C2, C3, C4, and C1-N, ensuring that all models and all three CPV lateralities within each outcome used identical participants, event indicators, and follow-up times. Cox regression coefficients and standard errors were combined using Rubin’s rules and exponentiated to obtain hazard ratios and 95% confidence intervals. As a sensitivity analysis, C1–C4 were also refitted without imputation in outcome-specific strict common complete-case samples. Missingness was reported for every covariate, and participants included in and excluded from the strict complete-case samples were compared using standardized differences.

The proportional-hazards assumption was evaluated using scaled Schoenfeld residuals in each imputed dataset. Model convergence and design-matrix rank were assessed. Influential observations were evaluated using DFBETA (the change in a regression coefficient after deletion of one observation), with an absolute threshold of 2/sqrt(n) used to identify potentially influential participants. Potential nonlinearity of CPV was examined using three-knot restricted cubic splines placed at the 10th, 50th, and 90th percentiles, and the nonlinear component was pooled across imputations using Rubin’s rules. Model calibration was evaluated at 3 and 5 years by comparing risks averaged across the 50 imputations with Kaplan–Meier estimates within predicted-risk quintiles. Calibration-in-the-large and inverse-probability-of-censoring-weighted Brier scores were calculated. Calibration slopes and Brier scores were internally validated using 500 participant-level bootstrap attempts distributed evenly across the completed datasets.

Longitudinal CPV trajectories were evaluated in the same fixed outcome-specific cohorts used for the Cox analyses. For converters, MRI examinations from the index MRI up to but not including the conversion date were retained; for non-converters, examinations through the censoring date were retained. Left, right, and bilateral CPV were residualized for ICV using the index-MRI transformation, standardized, and the same frozen transformation was applied to subsequent scans. Linear mixed-effects models included years since index MRI, standardized baseline log-HCY, their interaction, eventual conversion status, the time-by-conversion interaction, and C1 covariates, with participant-specific random intercepts and time slopes. Post-index HCY measurements were not used. Missing baseline HCY was imputed by predictive mean matching in 50 chained-equation datasets, whereas CPV, ICV, MRI dates, conversion status, and follow-up time were not imputed. Sensitivity analyses were restricted to participants with at least two eligible MRI examinations and to participants with observed temporally eligible HCY. FDR correction was applied across the three CPV lateralities within each cohort and trajectory term.

### 2.7. Single-Nucleus Reanalysis of CP Epithelial States

#### 2.7.1. Data Source

Publicly available single-nucleus RNA sequencing (snRNA-seq) data from postmortem human CP tissues were analyzed (Gene Expression Omnibus [GEO]: GSE315551; BioProject: PRJNA1398245). The original cohort included nine adult donors of lateral ventricular CP tissue, comprising four donors with AD and five controls [32]. However, the public metadata available through GEO, the Sequence Read Archive (SRA) RunInfo, and BioSample did not permit individual samples to be linked to their diagnostic groups. Donor-specific HCY concentrations and information on experimental HCY exposure were also unavailable. Accordingly, the transcriptomic data were analyzed independently of the ADNI imaging cohort and were used to characterize CP epithelial transcriptional states, predicted ligand–receptor interactions, and spatial organization. These analyses were considered exploratory and hypothesis-generating rather than mechanistic validation of the association between circulating HCY and CPV.

#### 2.7.2. snRNA-Seq Data Preprocessing

GSE315551 raw expression matrices were loaded into AnnData objects and merged with relevant GEO, SRA, and BioSample clinical metadata. Nuclei underwent quality control based on mitochondrial, ribosomal, and hemoglobin gene expression profiles. Post-QC data were normalized (10,000 counts/nucleus) and log1p-transformed; features expressed in fewer than three nuclei were discarded. Highly variable genes (HVGs; *n* = 3000) were selected using the Seurat-flavor method implemented in Scanpy 1.12.1, with sample ID specified as the batch key. The HVG matrix was corrected for sample-level batch effects using ComBat and subsequently scaled. Principal component analysis (PCA) was performed with 50 components, of which the first 40 were used to construct a 15-nearest-neighbor graph. Uniform Manifold Approximation and Projection (UMAP) was generated with min_dist = 0.35, and unsupervised Leiden clustering was performed at resolution 0.7 with random seed 0. The analysis included all 67,124 QC-qualified nuclei; the post-QC expression object contained 37,768 genes. A curated 48,309-nucleus object containing the populations retained for the original downstream analyses was subsequently generated; the module-ablation analysis used the full QC-qualified set.

#### 2.7.3. Cell Type Annotation and Epithelial State Definition

Cell identity was assigned by integrating Leiden clustering, UMAP localization, cluster-specific differential expression, and canonical markers. Key populations were annotated as follows: CP epithelial cells (TTR, AQP1, FOLR1, KRT8, KRT18, CLDN1, CLDN2); ciliated epithelial cells (FOXJ1, DNAH11, HYDIN, RSPH1, PIFO); endothelial cells (PECAM1, VWF, CLDN5, FLT1); fibroblasts/stromal cells (COL1A1, COL1A2, DCN, LUM); myeloid cells (C1QA, C1QB, C1QC, AIF1, CD68, TYROBP); and mural/pericyte/smooth muscle cells (SMCs) (PDGFRB, RGS5, ACTA2, TAGLN, MYH11). Additional identified populations included astroglia, neurons, and oligodendrocytes.

Within the CP epithelial compartment, we evaluated a curated one-carbon metabolism-related gene set spanning folate and one-carbon metabolism, homocysteine remethylation, methionine cycling, transsulfuration, and redox regulation (SLC19A1, FOLR1, MTR, MTRR, MTHFR, MTHFD1, MTHFD1L, BHMT2, CBS, CTH, MPST, GPX3, GPX4, SOD2, and NQO1). Leiden clusters 4 and 6, identified through unsupervised clustering, were designated one-carbon metabolism-enriched epithelial state A (OCM-Epi-A) and one-carbon metabolism-enriched epithelial state B (OCM-Epi-B), respectively, after post hoc evaluation of canonical epithelial markers and the curated one-carbon/HCY-related module. To evaluate module-independent recovery, the exact 123-gene module was removed before HVG selection. HVG selection (*n* = 3000), ComBat correction, scaling, PCA (50 components computed, with the first 40 used for neighborhood construction), construction of a 15-nearest-neighbor graph, UMAP (min_dist = 0.35), and Leiden clustering were then repeated on the 67,124 QC-qualified nuclei. Of the 123 module genes, 121 were detected and excluded. The primary analysis used seed 1337 and Leiden resolution 0.7, with prespecified sensitivity analyses across seeds 42, 1337, and 2026 and resolutions 0.6, 0.7, and 0.8. Recovery was quantified using cross-tabulation, adjusted Rand index (ARI), normalized mutual information (NMI), and state-specific F1 scores (harmonic means of precision and recall). These labels describe relative gene-expression patterns and do not indicate documented HCY exposure, responsiveness to elevated HCY, or involvement in HCY-induced pathology.

#### 2.7.4. Differential Expression Analysis of One-Carbon Metabolism-Enriched Epithelial States

To avoid treating individual nuclei as independent replicates, raw counts were aggregated by donor and cell state. OCM-Epi-A and OCM-Epi-B were separately compared with all other QC-passed nuclei using edgeR negative-binomial quasi-likelihood models with donor included as a blocking factor (~donor + condition). Only donors contributing both the target state and reference nuclei were included. Genes with insufficient expression were removed using filterByExpr, libraries were normalized using the trimmed mean of M values with singleton pairing (TMMwsp) method, and robust dispersion estimation was applied. Positive protein-coding markers were required to have a Benjamini–Hochberg-adjusted *p* < 0.05, log2 fold change > 0.5, and detection in >20% of target-state nuclei. Over-representation analyses used all protein-coding genes tested in the corresponding contrast as the background, with pathway *p* values adjusted by the Benjamini–Hochberg method within each state and gene-set library.

### 2.8. Spatial Transcriptomic Processing

#### 2.8.1. Data Preprocessing and Cross-Modality Label Transfer

Four spatial transcriptomic samples (GSE315553, generated via Curio Bioscience Seeker technology) were sourced from the same original study as the snRNA-seq dataset [32] and processed with their physical coordinates preserved. Raw counts were normalized (10,000 counts/spot) and log1p-transformed. Spatial transcriptomic data were analyzed using complementary proportion-estimation and label-transfer approaches. To estimate epithelial-state composition at the spot level, integrative and reference-informed tissue segmentation (IRIS) was applied. Genes and spots were retained using minCountGene = 1 and minCountSpot = 1, respectively, and the analysis was performed with numCluster = 6. The resulting IRIS estimates were interpreted as expression-derived mixing proportions within individual spatial spots and not as absolute cell numbers. Among the evaluated specifications, the CP-epithelial-only, Redox-excluded model with numCluster = 6 was selected for visualization because it showed the greatest between-spot variability.

In a separate analysis, reference cell-state labels from the single-nucleus RNA sequencing dataset were transferred to the spatial transcriptomic data using the ingest function in Scanpy. Each spatial spot was assigned a discrete reference label, and these assignments were subsequently used for spatial neighborhood-enrichment analysis in Squidpy. Spatial neighbor graphs were constructed using 30 neighbors per spot, and enrichment z-scores were calculated using 1000 permutations. Thus, the IRIS-derived proportions and Scanpy-ingest assignments were used for distinct analytical purposes.

To evaluate whether spatial label transfer depended on the curated module, Scanpy ingest was repeated using 1195 shared features with zero overlap with the 123-gene module and module-independent reference labels.

#### 2.8.2. Spatial Epithelial State Scoring

To address the limitations of hard-labeling for mixed or rare populations, continuous gene-set scoring was performed using Scanpy. Marker modules for Ciliated CP, Core CP, OCM-Epi-A/B, and Redox CP were scored and z-standardized per sample. These scores were used to characterize spatial variation in the epithelial states and to assess whether hard-label assignment underestimated rare or mixed states.

#### 2.8.3. Spatial Co-Localization Analysis

Neighborhood enrichment was computed using Squidpy with 1000 permutations. Spatial adjacency graphs were built by assigning 30 physical neighbors to each spot. The resulting z-scores were used to identify state pairs that co-localized more frequently than expected by chance. The analysis focused on spatial neighborhood enrichment between OCM-Epi-A/B–like spots and endothelial, myeloid, stromal, and perivascular populations. Robust interactions required a median z-score ≥ 2 across at least three samples, with rare-label interactions categorized as exploratory.

#### 2.8.4. Ligand–Receptor Interaction Analysis

Potential ligand–receptor interactions among the annotated cell populations were evaluated using the LIgand–receptor ANalysis frAmework (LIANA) implementation of the CellChat scoring method and the CellChatDB ligand–receptor resource. Ligand–receptor pairs were required to be expressed in at least 5% of cells in the relevant cell group, and groups containing fewer than 20 cells were excluded. Statistical support was evaluated using 1000 permutations with a random seed of 1337. Communication scores were log1p-transformed for visualization. These results were interpreted as expression-based predictions of potential intercellular communication rather than direct evidence of signaling activity.

### 2.9. Statistical Analysis

Continuous variables were summarized as means with standard deviations or medians with interquartile ranges, and categorical variables as counts and percentages. Unless otherwise specified, all tests were two-sided; nominal statistical significance was defined as *p* < 0.05, and adjusted statistical significance was defined as an FDR < 0.05 after Benjamini–Hochberg correction within each prespecified testing family. Diagnostic-stage analyses used Kruskal–Wallis tests, pairwise Mann–Whitney U tests, and multinomial logistic regression. Structural MRI phenotypes were analyzed using ordinary least-squares regression with HC3 robust standard errors. The primary MRI screening family comprised the 42 index-HCY/C1 phenotype tests. C2–C4 estimates for phenotypes selected in C1 were treated as sensitivity analyses, whereas C1-N and each repeated-HCY definition used their separately prespecified testing families. Diagnostic-stage and structural MRI analyses used complete-case data; the C1–C4 MRI models were fitted in the same 709 participants.

Associations between CPV and diagnostic progression were estimated using Cox proportional-hazards models in fixed outcome-specific cohorts. Covariate specifications for C1–C4, C1-N, S1, and S2 are summarized in Table 1. Missing non-outcome adjustment covariates were imputed in 50 chained-equation datasets, estimates were pooled using Rubin’s rules, and strict common complete-case analyses were performed as sensitivity analyses. Benjamini–Hochberg correction was applied across the three CPV lateralities within each outcome and model. The proportional-hazards assumption, influential observations, and nonlinearity were assessed using scaled Schoenfeld residuals, DFBETA, and restricted cubic splines, respectively. Calibration and discrimination were evaluated using calibration-in-the-large, calibration slopes, inverse-probability-of-censoring-weighted Brier scores, Harrell’s C-index, and 3- and 5-year time-dependent areas under the curve, with participant-level bootstrap validation. Longitudinal CPV trajectories were assessed using linear mixed-effects models with participant-specific random intercepts and slopes; Benjamini–Hochberg correction was applied across the three CPV lateralities within each cohort and trajectory term.

For the transcriptomic analyses, donor-aggregated pseudobulk counts were analyzed using edgeR negative-binomial quasi-likelihood models with donor blocking. Benjamini–Hochberg correction was applied within each state-level gene contrast and within each state-by-gene-set-library enrichment family. Module-independent clustering stability was summarized using the adjusted Rand index, normalized mutual information, and state-specific F1 scores. Spatial neighborhood enrichment and ligand–receptor predictions were evaluated using 1000 permutations in Squidpy and LIANA, respectively; patterns represented in only a limited number of spatial samples were treated as exploratory. Analyses were conducted in Python and R using pandas 3.0.0, NumPy 2.5.1, SciPy 1.17.0, statsmodels 0.14.6, Matplotlib 3.11.0, Seaborn 0.13.2, Scanpy, AnnData, LIANA, Squidpy, edgeR, and ggplot2. The transcriptomic reanalysis used Python 3.13.5, Scanpy 1.12.1, AnnData 0.12.10, and LIANA 1.8.1.

## 3. Results

### 3.1. Higher HCY Levels Are Observed Across the AD Clinical Spectrum but Show Limited Associations with Clinical Progression

**Figure 1:**
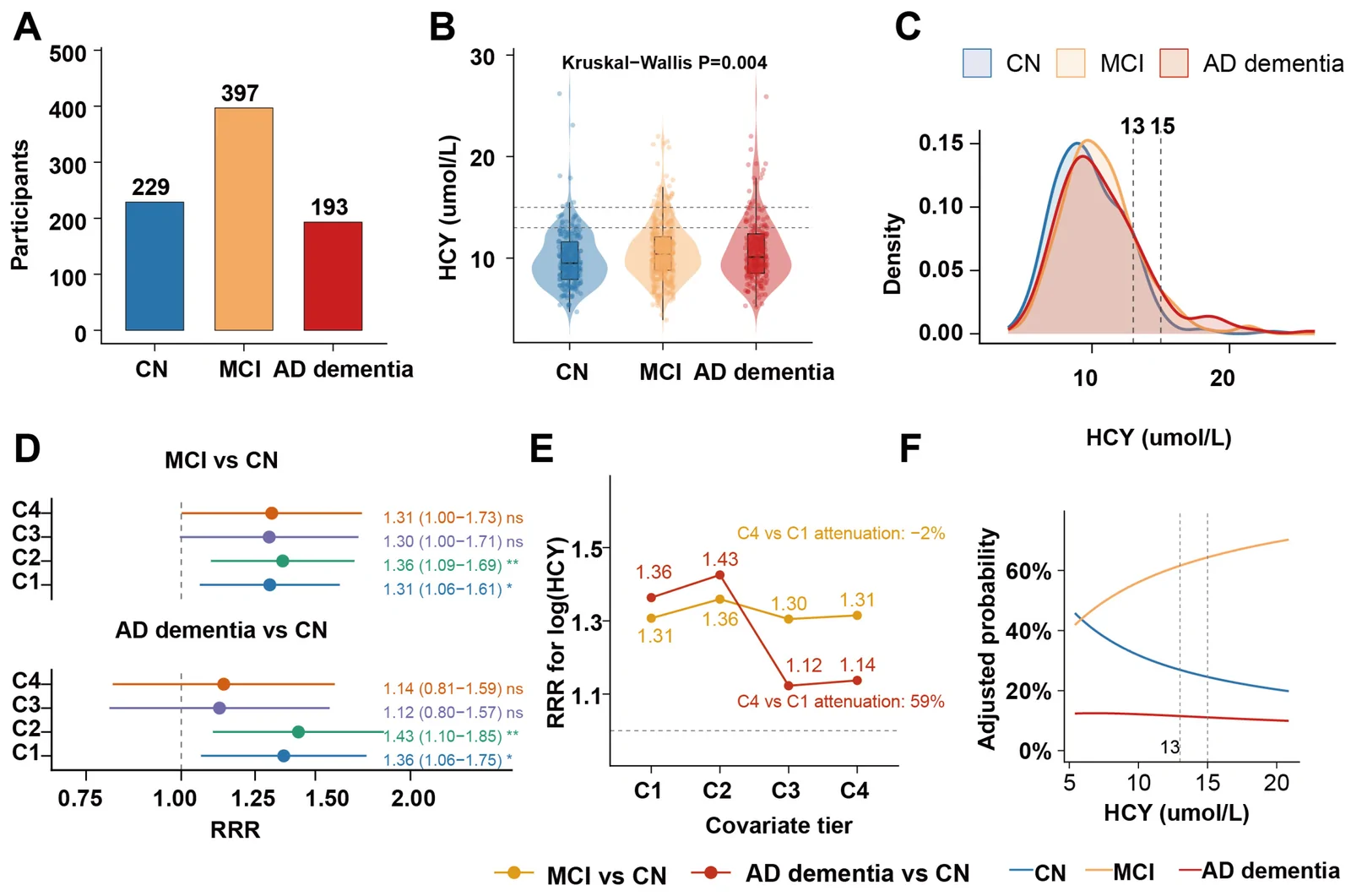
Baseline HCY enrichment across the AD diagnostic continuum. (**A**) Distribution of baseline diagnostic groups in the analysis cohort after HCY quality control. (**B**) Baseline plasma HCY concentrations across the CN, MCI, and AD dementia groups. Violin plots show the distribution of raw HCY values with embedded boxplots; the global Kruskal–Wallis test was used to compare diagnostic groups. Dashed horizontal lines indicate clinically used HCY thresholds of 13 and 15 μmol/L. (**C**) Density overlap of raw HCY values by diagnostic group, with dashed vertical lines marking 13 and 15 μmol/L. (**D**) Multinomial logistic regression estimates for the association between 1-SD higher log-transformed HCY and diagnostic stage, using CN as the reference group. Relative risk ratios (RRRs) and 95% confidence intervals are shown across sequential covariate tiers C1–C4. C1 adjusted for standard clinical covariates; C2 further adjusted for vascular and comorbidity variables; C3 further adjusted for laboratory variables; and C4 further adjusted for medication variables. Significance annotations reflect FDR-adjusted *p* values (* P_FDR < 0.05, ** P_FDR < 0.01, ns not significant). (**E**) Covariate-tier trajectory of RRRs for MCI versus CN and AD dementia versus CN, illustrating attenuation after laboratory and medication adjustment. C4 versus C1 attenuation was calculated on the log-RRR scale. (**F**) Adjusted diagnostic probabilities across the observed HCY range, derived from the multinomial logistic model, with 13 and 15 μmol/L thresholds indicated. Overall, higher HCY was enriched in MCI and AD dementia at baseline, but the AD dementia contrast was attenuated after broader laboratory and medication adjustment.

The baseline diagnostic-stage cohort included 229 CN participants, 397 participants with MCI, and 193 participants with AD dementia (Figure 1A). HCY concentrations differed across diagnostic groups (Kruskal–Wallis *p* = 0.004; Figure 1B). The distribution curves showed broad overlap among CN, MCI, and AD dementia, with reference lines at 13 and 15 μmol/L crossing the upper portion of all three distributions (Figure 1C).

In multinomial models using CN as the reference group, each 1-SD increase in log-transformed HCY was associated with higher relative risk ratios for MCI across the covariate tiers (C1 RRR = 1.31, 95% CI 1.06–1.61; C2 RRR = 1.36, 95% CI 1.09–1.69; C3 RRR = 1.30, 95% CI 1.00–1.71; C4 RRR = 1.31, 95% CI 1.00–1.73; Figure 1D). For AD dementia versus CN, the association was present in C1 and C2 but attenuated after laboratory and medication adjustment (C1 RRR = 1.36, 95% CI 1.06–1.75; C2 RRR = 1.43, 95% CI 1.10–1.85; C3 RRR = 1.12, 95% CI 0.80–1.57; C4 RRR = 1.14, 95% CI 0.81–1.59; Figure 1D). The covariate-tier trajectory showed that the MCI-versus-CN RRR remained similar from C1 to C4 (1.31, 1.36, 1.30, and 1.31), whereas the AD dementia-versus-CN RRR declined from 1.36 in C1 to 1.14 in C4, corresponding to 59% attenuation (Figure 1E). In the adjusted probability curves, the estimated probability of CN decreased as HCY increased, the estimated probability of MCI increased over the same range, and the estimated probability of AD dementia changed comparatively little; the 13 and 15 μmol/L reference lines fell within the rising portion of the MCI curve (Figure 1F).

### 3.2. Higher Index HCY Is Associated with Larger Choroid Plexus Volume and Lower Temporolimbic Structural Measures

**Figure 2:**
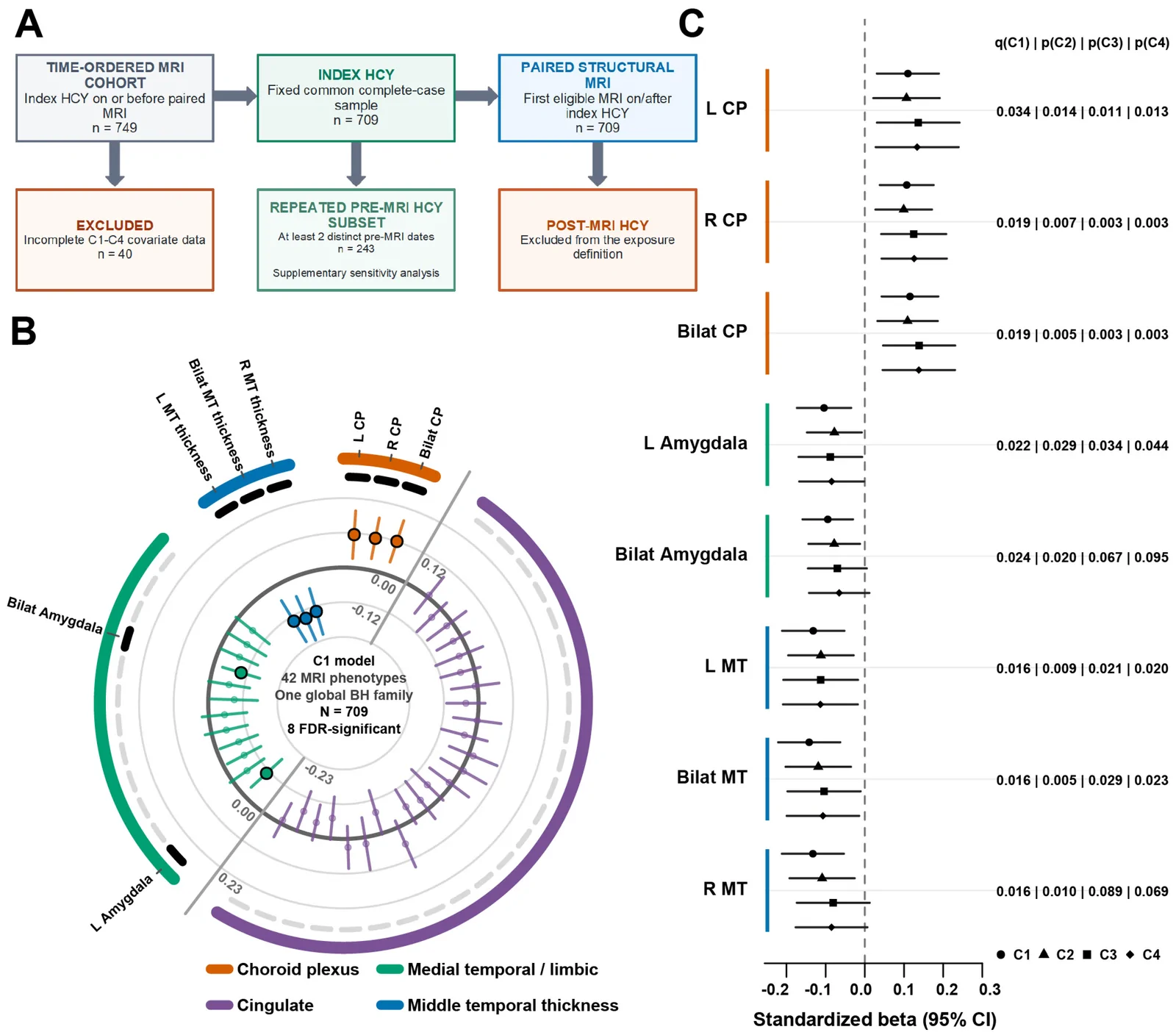
Associations between index homocysteine and structural magnetic resonance imaging phenotypes. (**A**) Temporal ordering and selection of the analytic samples. (**B**) Associations of standardized index log–HCY with 42 structural MRI phenotypes in C1. Points and radial lines represent standardized β coefficients and 95% confidence intervals (CIs), respectively. Colors indicate anatomical categories, and the bold black ring denotes β = 0. Black outer segments and enlarged outlined points indicate associations significant after false discovery rate (FDR) correction. (**C**) Effect estimates for the eight C1–selected phenotypes across C1–C4. Circles, triangles, squares, and diamonds represent C1, C2, C3, and C4, respectively; horizontal lines represent 95% CIs, and the vertical dashed line denotes β = 0. Abbreviations: Bilat, bilateral; CI, confidence interval; CP, choroid plexus; FDR, false discovery rate; HCY, homocysteine; L, left; MRI, magnetic resonance imaging; MT, middle temporal; R, right.

Application of the prespecified temporal-ordering criteria yielded 749 participants whose index homocysteine (HCY) measurement preceded or coincided with the paired magnetic resonance imaging (MRI) examination. Among them, 709 had complete data for all covariates included in C1–C4 and constituted the fixed common complete-case sample used across the sequential models (Figure 2A; Supplementary Table S1).

In C1, standardized index log-HCY was examined in relation to 42 prespecified structural MRI phenotypes, with Benjamini–Hochberg correction applied across all phenotypes as a single testing family. Eight phenotypes remained significant after false discovery rate (FDR) correction (Figure 2B; Supplementary Table S2). Higher index HCY was associated with larger left choroid plexus volume (CPV; β = 0.110, 95% confidence interval [CI]: 0.031 to 0.189, *p* = 0.006, FDR = 0.034), right CPV (β = 0.107, 95% CI: 0.038 to 0.176, *p* = 0.002, FDR = 0.019), and bilateral CPV (β = 0.115, 95% CI: 0.043 to 0.188, *p* = 0.002, FDR = 0.019) (Figure 2B; Supplementary Table S2). Inverse associations were observed for left and bilateral amygdala volumes (β = −0.104 and −0.095; FDR = 0.022 and 0.024, respectively) and for left, right, and bilateral middle temporal thickness (β = −0.132, −0.133, and −0.142; FDR = 0.016 for all three measures) (Figure 2B; Supplementary Table S2).

To assess the robustness of the C1 findings, the eight MRI phenotypes that remained significant after global FDR correction were further evaluated in progressively adjusted C2–C4 sensitivity models using the same 709 participants, and their effect estimates were compared across models. The estimates for CPV remained positive throughout the sequential adjustment. In C4, the estimates were β = 0.134 (95% CI: 0.028 to 0.240, *p* = 0.013) for left CPV, β = 0.126 (95% CI: 0.043 to 0.209, *p* = 0.003) for right CPV, and β = 0.138 (95% CI: 0.045 to 0.230, *p* = 0.003) for bilateral CPV (Figure 2C; Supplementary Table S2). The inverse estimates for left and bilateral middle temporal thickness were also maintained after progressive adjustment, whereas the estimate for right middle temporal thickness was attenuated. The estimate for left amygdala volume remained inverse across C2–C4, whereas that for bilateral amygdala volume was attenuated in the more extensively adjusted models (Figure 2C).

In the independent C1-N neuroimaging sensitivity analysis, same-sample C1 and C1-N each included 747 participants for all three CPV measures. Additional adjustment for scanner batch, field strength, lateral ventricular volume, total CSF volume, total gray matter volume, and hippocampal volume attenuated but did not eliminate the positive associations for left CPV (β = 0.106, 95% CI 0.023–0.190), right CPV (β = 0.093, 95% CI 0.024–0.162), and bilateral CPV (β = 0.108, 95% CI 0.033–0.183); all three C1-N associations had FDR = 0.012. Relative to same-sample C1, the beta estimates were attenuated by 14.6%, 21.0%, and 17.0%, respectively.

In the smaller repeated-measurement subset (n = 243), none of the associations of the mean, maximum, or time-normalized area under the curve of pre-MRI log-HCY with the 42 MRI phenotypes remained significant after FDR correction (Supplementary Table S3).

### 3.3. CPV Shows Model-Dependent Associations with MCI-to-AD Dementia Progression

The multiple-imputation Cox analyses included 177 participants with 55 events in the CN-to-MCI cohort and 297 participants with 164 events in the MCI-to-AD dementia cohort. Within each outcome, C1–C4, C1-N, and all three CPV lateralities used identical participants, event indicators, and follow-up times. Body mass index (BMI) was the most frequently missing covariate, followed by thyroid-stimulating hormone (TSH) and vitamin B12; complete covariate-specific missingness and comparisons between included and excluded participants are provided in Supplementary Table S4. The strict common complete-case sensitivity analyses included 158 participants with 48 events for CN-to-MCI and 255 participants with 141 events for MCI-to-AD dementia.

**Figure 3:**
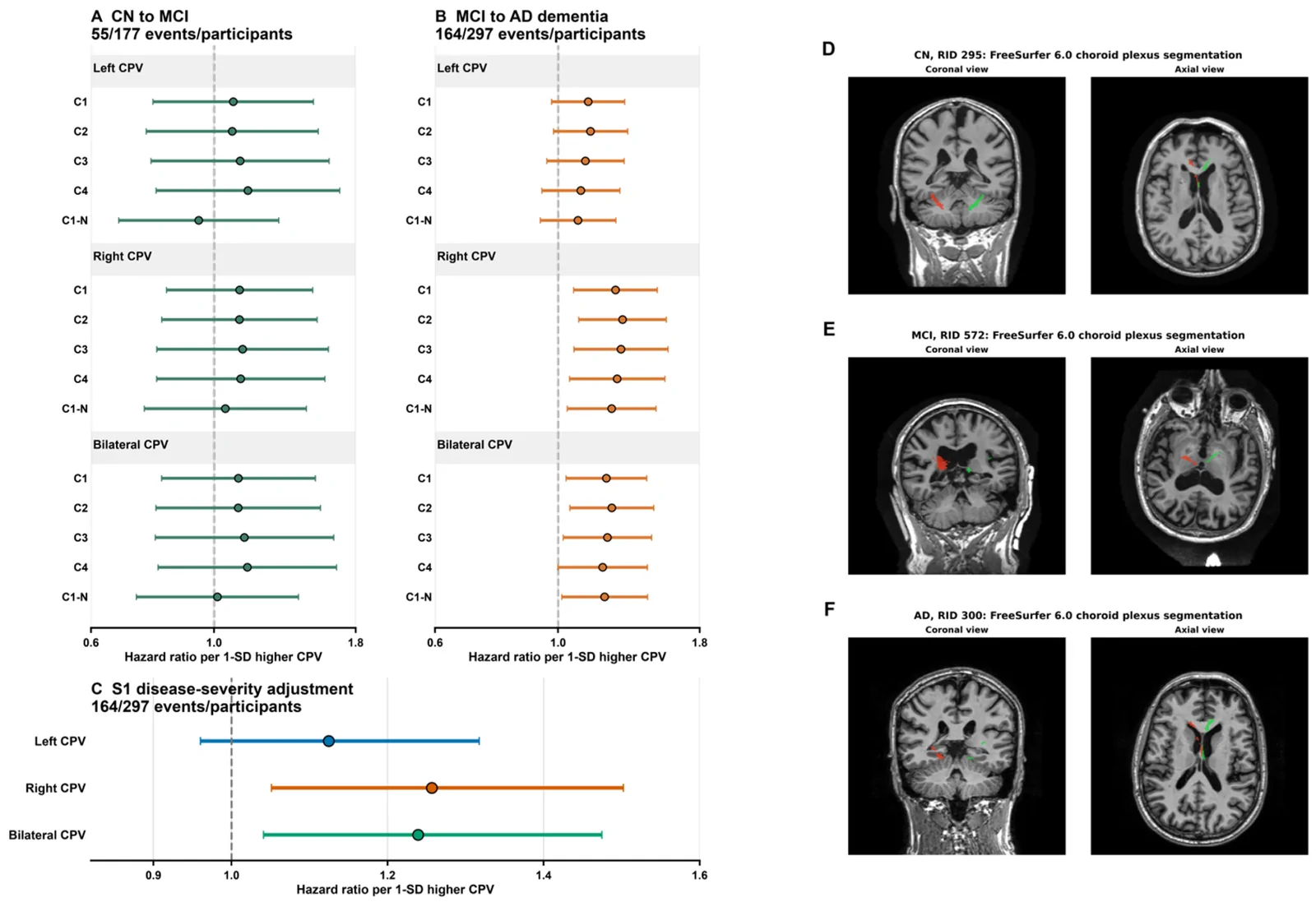
Choroid plexus volume, clinical progression, disease-severity sensitivity analysis, and representative segmentations. Cox proportional hazards models estimated the association of each 1-SD increase in intracranial-volume-residualized left, right, or bilateral choroid plexus volume (CPV) with (**A**) CN-to-MCI progression and (**B**) MCI-to-AD dementia progression. Within each endpoint, C1–C4 and C1-N used identical participants, events, and follow-up times; missing non-outcome adjustment covariates were multiply imputed in 50 datasets and estimates were combined using Rubin’s rules. (**C**) The post hoc S1 disease-severity model was fitted in the fixed MCI-to-AD dementia cohort and additionally adjusted for CDR-SB, scanner batch, laterality-matched hippocampal and entorhinal measures, and white matter hypointensity burden. Points and horizontal lines in (**A**–**C**) indicate hazard ratios and 95% confidence intervals. (**D**–**F**) Representative MRI illustrations of the CP labels are shown for CN (participant 295), MCI (participant 572), and AD dementia (participant 300), respectively. Green overlays indicate Left-choroid-plexus (label 31), and red overlays indicate Right-choroid-plexus (label 63), shown in coronal and axial views. The segmentation panels illustrate anatomical label localization, whereas all quantitative model estimates were derived from the PHC-released scalar CPV measures. CN, cognitively normal; MCI, mild cognitive impairment; AD, Alzheimer’s disease.

In the CN-to-MCI cohort, none of the left, right, or bilateral CPV measures was associated with progression across C1–C4 or C1-N (hazard ratio [HR] range, 0.94–1.15; all FDR ≥ 0.517; Figure 3A). In contrast, higher right CPV was associated with MCI-to-AD dementia progression in C1 (HR = 1.27, 95% CI 1.07–1.51; FDR = 0.023), C2 (HR = 1.31, 95% CI 1.09–1.57; FDR = 0.013), C3 (HR = 1.30, 95% CI 1.07–1.58; FDR = 0.028), and C4 (HR = 1.28, 95% CI 1.05–1.56; FDR = 0.047; Figure 3B). The C1-N estimate was similar in direction and magnitude (HR = 1.25, 95% CI 1.04–1.50; nominal *p* = 0.019), but did not meet the FDR threshold (FDR = 0.051).

Bilateral CPV was associated with MCI-to-AD dementia progression in C1 (HR = 1.22, 95% CI 1.03–1.44; FDR = 0.029), C2 (HR = 1.25, 95% CI 1.05–1.49; FDR = 0.019), and C3 (HR = 1.23, 95% CI 1.02–1.47; FDR = 0.044). The bilateral association did not meet the FDR threshold in C4 (HR = 1.20, 95% CI 1.00–1.45; nominal *p* = 0.051; FDR = 0.077) or C1-N (HR = 1.21, 95% CI 1.01–1.45; nominal *p* = 0.034; FDR = 0.051). Left CPV was not associated with MCI-to-AD dementia progression in any model. Overall, the positive estimates for right and bilateral CPV varied across covariate and sensitivity models, indicating that the prognostic evidence was not uniformly robust. The observed side-specific pattern was treated as descriptive and was not interpreted as established biological lateralization.

The strict common complete-case analysis produced no evidence of an association between CPV and CN-to-MCI progression. In the MCI-to-AD dementia cohort, left, right, and bilateral CPV showed positive associations in C1–C3, but none met the FDR threshold in C4 (FDR = 0.091, 0.087, and 0.087, respectively; Supplementary Table S4). Thus, the attenuation in C4 was also observed when all covariate tiers were compared in the same non-imputed participants.

In the S1 disease-severity analysis, right CPV (HR = 1.26, 95% CI 1.05–1.50; FDR = 0.024) and bilateral CPV (HR = 1.24, 95% CI 1.04–1.47; FDR = 0.024) retained positive prognostic associations, whereas left CPV did not (HR = 1.12, 95% CI 0.96–1.32; FDR = 0.144; Figure 3C). Representative MRI illustrations of the CP labels are shown for CN, MCI, and AD dementia in Figure 3D–F, respectively. In the accompanying incremental-prediction analysis, adding CPV to S1 did not clearly improve discrimination: the 95% bootstrap intervals for optimism-corrected changes in the C-index and 3- and 5-year time-dependent AUC all included zero (Supplementary Figure S1A). Thus, CPV showed model-dependent prognostic associations but did not demonstrate clear incremental predictive value.

The exploratory S2 analysis included 62 participants with observed pre-index CSF amyloid-beta 42 and phosphorylated tau (36 conversion events); none of the CPV associations was statistically significant after molecular-biomarker adjustment (Supplementary Figure S1B; Supplementary Table S5). Incremental prediction likewise showed no clear improvement, with all 95% bootstrap intervals for changes in the C-index and 3- and 5-year time-dependent AUC including zero (Supplementary Figure S1C).

Across the primary progression models, mean predicted risks were close to the corresponding Kaplan–Meier estimates at 3 and 5 years, with maximum absolute calibration-in-the-large differences below 0.01 in both progression cohorts. However, optimism-corrected calibration slopes were below 1 and decreased with model complexity. In the MCI-to-AD dementia cohort, slopes were 0.80–0.82 in C1 and 0.44–0.49 in C4. Bootstrap validation of the CN-to-MCI C4 models was unstable because only 30–34 of 500 attempts produced estimable calibration results (Supplementary Figure S2; Supplementary Table S6).

Restricted cubic-spline analyses provided no evidence of CPV nonlinearity in the CN-to-MCI cohort. In the MCI-to-AD dementia cohort, nominal nonlinearity was observed only for left CPV in C3 and C4 (*p* = 0.021 and *p* = 0.024), whereas right and bilateral CPV showed no evidence of nonlinearity across the covariate tiers (Supplementary Figure S3; Supplementary Table S6). Scaled Schoenfeld residuals indicated non-proportional CPV effects in several MCI-to-AD models, particularly for right and bilateral CPV; the reported Cox hazard ratios should therefore be interpreted as average associations over follow-up. In the S1 time-varying sensitivity analysis, the right and bilateral associations were stronger at 1 year and approached the null by 5 years. All primary models converged, and no observation exceeded the prespecified DFBETA influence threshold.

Longitudinal MRI analyses included the fixed CN-to-MCI cohort of 177 participants with 55 conversions (1031 pre-endpoint MRI examinations) and the fixed MCI-to-AD dementia cohort of 297 participants with 164 conversions (1207 examinations). Mean ICV-residualized CPV increased modestly over follow-up in both cohorts (CN-to-MCI: β = 0.022–0.037 SD/year, all FDR ≤ 0.002; MCI-to-AD dementia: β = 0.035–0.047 SD/year, all FDR ≤ 0.003). However, baseline log-HCY was not associated with a faster CPV trajectory in either cohort (all HCY-by-time FDR ≥ 0.203), and pre-conversion CPV slopes did not differ between converters and non-converters (all conversion-by-time FDR ≥ 0.929; Supplementary Figure S4 and Supplementary Table S7). The findings were materially unchanged in sensitivity analyses restricted to participants with repeated MRI examinations and in those restricted to participants with observed HCY.

### 3.4. Single-Nucleus Transcriptomic Analysis Identifies One-Carbon Metabolism-Enriched Choroid Plexus Epithelial States

**Figure 4:**
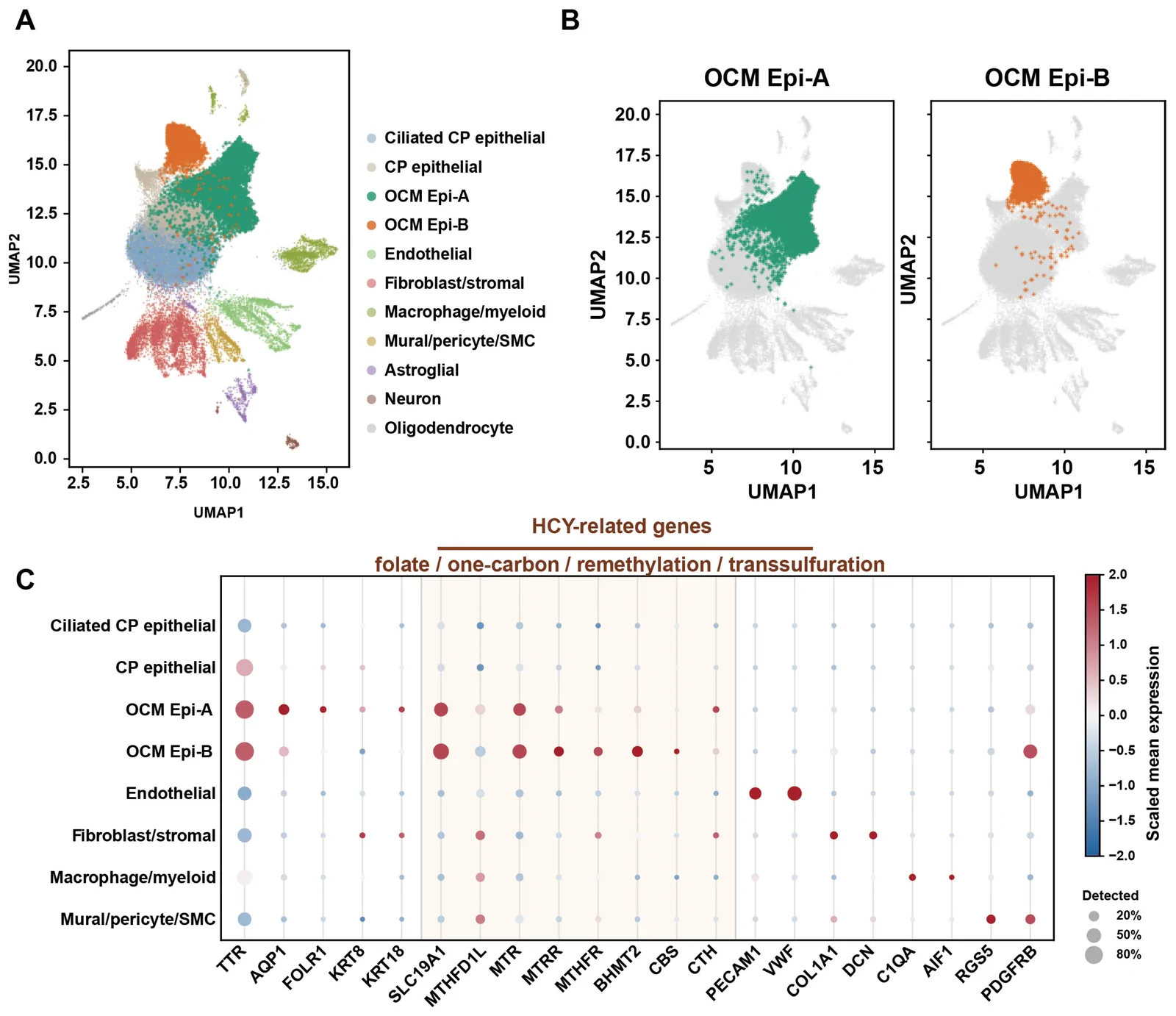
Identification of one-carbon metabolism-enriched epithelial states in the human CP. (**A**) Uniform Manifold Approximation and Projection (UMAP) visualization of snRNA-seq from human CP, colored by annotated cell type. CP epithelial, ciliated epithelial, endothelial, fibroblast/stromal, macrophage/myeloid, mural/pericyte/smooth muscle, astroglial, neuronal, and oligodendrocyte populations are shown. (**B**) UMAP highlighting the two epithelial states with relatively high expression of genes in the curated one-carbon metabolism-related module, designated OCM-Epi-A and OCM-Epi-B. Gray points indicate all other nuclei. OCM-Epi-A and OCM-Epi-B are shown in green and red, respectively. (**C**) Dot plot showing expression of epithelial markers, genes in the curated one-carbon metabolism-related module, and representative lineage markers across annotated cell populations. Dot color represents scaled mean expression, and dot size represents the percentage of nuclei with detectable expression. The shaded region marks the curated one-carbon metabolism-related gene set, including genes involved in folate transport, one-carbon metabolism, remethylation, and transsulfuration. The OCM-Epi-A/B labels denote relative gene-expression enrichment and do not imply documented HCY exposure or responsiveness. OCM-Epi-A and OCM-Epi-B were derived from unsupervised Leiden clusters and annotated post hoc; module-exclusion recovery and donor representation are presented in Supplementary Figure S5.

To characterize CP epithelial expression states that could provide biological context for the imaging findings, unsupervised Leiden clustering followed by post hoc annotation identified two epithelial states with relatively high expression of the curated one-carbon/HCY-related module, designated OCM-Epi-A and OCM-Epi-B (Figure 4A–C). After the curated 123-gene one-carbon/HCY-related module was excluded before HVG selection and reclustering, both states remained distinguishable (state-specific F1 = 0.943 and 0.803, respectively; global ARI = 0.642; NMI = 0.740), However, donor representation was uneven. OCM-Epi-A was concentrated in A.3, A.4, and YA.2, whereas 93.6% of the original OCM-Epi-B nuclei were derived from YA.1 (Supplementary Figure S5).

In donor-level pseudobulk differential-expression analyses comparing each epithelial state with all other QC-passed nuclei while including donor as a blocking factor, 70 protein-coding genes met the prespecified marker criteria for OCM-Epi-A, whereas no protein-coding gene met all criteria for OCM-Epi-B. The only Epi-B gene satisfying the gene-level statistical, fold-change, and detection thresholds was the non-coding RNA SAMMSON, which was excluded by the protein-coding criterion. Across Gene Ontology (GO) Biological Process, Reactome, Kyoto Encyclopedia of Genes and Genomes (KEGG), and Hallmark analyses, no pathway met FDR < 0.05 for either state. Five Epi-A KEGG terms met nominal *p* < 0.05, but none survived multiplicity correction and they were not interpreted as enriched pathways. Accordingly, these analyses did not support a stable state-specific functional interpretation (Supplementary Table S8).

### 3.5. Spatial Mapping and Ligand–Receptor Analysis Characterize the Microenvironment of One-Carbon Metabolism-Enriched Epithelial States

**Figure 5:**
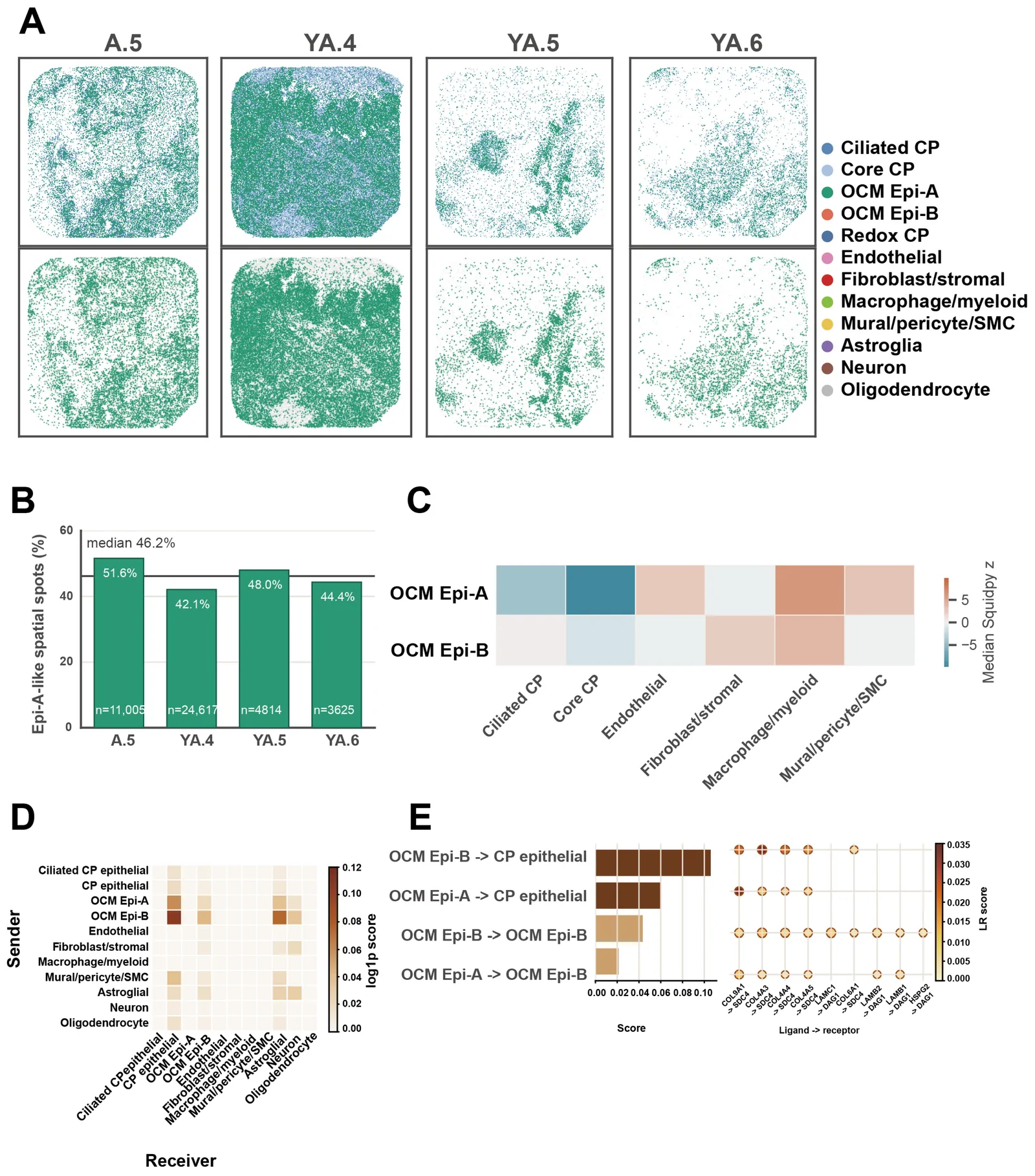
Spatial distribution and predicted expression-based interactions of one-carbon metabolism-enriched CP epithelial states. (**A**) IRIS-mapped spatial distribution of the Epi-A-like state. Each dot represents a spatial transcriptomic spot, and the color indicates the IRIS-estimated OCM-Epi-A proportion in that spot, with darker colors representing a stronger Epi-A-like signature. These proportions denote expression mixing weights inferred by IRIS and are not equivalent to actual cell counts within individual spots. (**B**) The mean IRIS-estimated OCM-Epi-A proportion was calculated across spatial spots within each sample. (**C**) Spatial neighborhood enrichment heatmap showing median Squidpy z-scores between one-carbon metabolism-enriched epithelial states and selected neighboring cell types. Higher positive z-scores indicate stronger spatial neighborhood enrichment, whereas negative values indicate reduced neighborhood enrichment. Estimates available in fewer than three spatial samples were treated as exploratory; the Epi-A–macrophage/myeloid estimate was available in one sample. (**D**) Heatmap of expression-based predicted ligand–receptor interaction scores across annotated sender and receiver cell populations in the single-nucleus dataset. Values are shown as log1p-transformed communication scores. (**E**) Predicted ligand–receptor interactions. The left bar plot summarizes the highest-scoring epithelial sender–receiver pairs, and the right dot plot shows representative ligand–receptor pairs contributing to these interactions. Dot color indicates the ligand–receptor score.

To characterize the spatial distribution of these module-enriched states, we projected the snRNA-seq-derived OCM-Epi-A and OCM-Epi-B signatures onto CP spatial transcriptomic sections. Under the CP-epithelial-only, Redox-excluded IRIS reference, mean spot-level mixing proportions were narrowly distributed across the four spatial libraries for OCM-Epi-A (23.66–24.04%) and OCM-Epi-B (20.18–20.82%; Supplementary Figure S5F); the OCM-Epi-A spatial distribution is shown in Figure 5A,B.

To assess spatial co-occurrence patterns independently of the IRIS-derived proportion estimates, we performed Squidpy neighborhood-enrichment analysis using the discrete spot labels assigned by Scanpy ingest. OCM-Epi-A–like spots showed cross-sample neighborhood enrichment with endothelial spots across the four spatial samples (median z = 2.74; z > 2 in three samples). A positive neighborhood association with macrophage/myeloid spots was observed in the only evaluable sample (z = 6.14) and was therefore considered exploratory rather than cross-sample robust (Figure 5C).

The module-independent ingest sensitivity analysis recovered both corresponding spatial states, although OCM-Epi-B assignments were rare.

To examine potential expression-based intercellular interactions, we applied ligand–receptor analysis to the single-nucleus dataset. Among the predicted interactions involving OCM-Epi-A, interactions with OCM-Epi-B received relatively high scores, with COL9A1–SDC4 among the highest-ranked ligand–receptor pairs. This analysis identifies COL9A1–SDC4 as a candidate expression-based ECM-related ligand–receptor pair between the two epithelial states.

## 4. Discussion

This study evaluated circulating HCY, CPV, and disease progression using clinical and structural MRI data from ADNI. In a separate exploratory analysis, we reanalyzed human CP single-nucleus and spatial transcriptomic datasets from an independent postmortem cohort. Elevated HCY levels were associated with both MCI and AD dementia, but the MCI association was less attenuated than the AD dementia association across the covariate-adjustment tiers. In a phenotype-wide screening of MRI-derived structural features, CPV emerged as one of the few HCY-associated signals that persisted after comprehensive covariate adjustment. Prospective conversion analyses based on index CPV showed model-dependent associations of right and bilateral CPV with MCI-to-AD dementia progression. The transcriptomic analyses identified one-carbon metabolism-enriched CP epithelial expression states and described their spatial organization. These findings provide hypothesis-generating tissue-level biological context for the ADNI imaging associations. Together, the cohort analyses support parallel associations of circulating HCY with CPV and of CPV with MCI-to-AD dementia progression, whereas the transcriptomic findings identify epithelial expression states that require further validation.

Our findings support previous observations regarding the association between HCY and cognitive impairment. Epidemiological studies and meta-analyses have demonstrated that elevated HCY levels are associated with an increased risk of cognitive decline, MCI, dementia, and AD [33,34], with AD patients exhibiting significantly higher mean HCY levels than healthy controls. In parallel, some studies have reported a positive correlation between HCY levels and disease severity [35], suggesting a potential role for HCY in the progression of the AD continuum. However, our analysis reveals that this association is not uniform across different disease stages. Although participants with MCI and AD dementia showed elevated HCY levels, the association between HCY and AD was substantially attenuated after stepwise adjustment for renal function, vitamin B12, lipid profiles, and medication use, whereas the association with MCI remained relatively stable. This finding suggests that HCY may primarily reflect pathological processes operating during earlier symptomatic stages rather than late-stage neurodegeneration per se. Moreover, HCY levels are influenced by multiple factors including folate status, vitamin B12 levels, renal function, and various comorbidities [36], making them more susceptible to confounding by complex clinical backgrounds in later disease stages. Observational neuroimaging evidence links higher HCY to greater white-matter lesion burden [37]. Experimental studies further indicate that HCY can induce oxidative stress and disrupt endothelial endoplasmic-reticulum redox homeostasis [38,39]. This interpretation is also informed by randomized controlled trials. B-vitamin treatment slowed brain atrophy in older adults with MCI [10], whereas high-dose B vitamins did not slow cognitive decline in patients with mild-to-moderate AD [11]. Similarly, although B-vitamin interventions effectively reduced circulating HCY levels, they generally failed to produce meaningful improvements in global cognitive performance, as assessed by measures such as the MMSE [40,41]. Taken together, these observations suggest that the contribution of HCY may be most pronounced during the early symptomatic stages of the AD continuum, before clinical manifestations become predominantly driven by established neurodegenerative pathology.

One notable finding was that CPV was among the HCY-associated imaging phenotypes retained after the prespecified global C1 screening and continued to show positive estimates across the progressively adjusted sensitivity models. Most associations involving canonical neurodegenerative regions were attenuated after laboratory and medication adjustment, whereas the positive CPV estimates were comparatively persistent. These findings identify CPV as a cross-sectional correlate of index HCY within the present analysis rather than establishing it as the most robust imaging marker or as a consequence of long-term HCY exposure. Recent neuroimaging studies have consistently reported enlarged CPV in patients with AD compared with cognitively normal older adults or individuals with subjective cognitive decline [18,19,20,21]. Moreover, greater CPV has been associated with poorer cognitive performance and accelerated longitudinal decline in MMSE scores [20]. From a biological perspective, the persistent association between HCY and CPV is plausible. As the principal component of the blood–cerebrospinal fluid barrier, the choroid plexus plays essential roles in cerebrospinal fluid (CSF) production, folate transport [42], metabolite exchange [16], immune surveillance [43], and maintenance of central nervous system homeostasis [44]. Notably, both folate and HCY are integral components of one-carbon metabolism, and previous studies have reported reduced cerebrospinal fluid folate concentrations [24] accompanied by elevated plasma HCY levels [45] in patients with AD. Given the role of the choroid plexus in regulating folate transport and metabolic exchange, it represents a plausible target of HCY-related metabolic dysregulation. Longitudinal MRI analyses showed modest average increases in CPV over follow-up but no detectable interaction between baseline HCY and time. The cross-sectional HCY–CPV association and longitudinal CPV trajectory should therefore be interpreted as complementary but distinct findings, without evidence that higher baseline HCY accelerates CPV enlargement. Longitudinal AD biomarker studies have identified distinct temporal inflection points across the disease continuum [46], underscoring the need for aligned repeated measurements to clarify the temporal relationship between HCY and CPV.

The progression analyses collectively support CPV as an exploratory prognostic correlate rather than a consistently independent predictor. Consistent with this cautious interpretation, pre-conversion CPV slopes did not differ between converters and non-converters, suggesting that the observed prognostic signal was related to index CPV level rather than a detectable conversion-specific rate of CPV enlargement. Although right CPV remained associated through C4 in the multiple-imputation analysis, the bilateral estimate was attenuated in C4, and none of the three CPV measures met the C4 FDR threshold in the strict common complete-case analysis. The absence of detectable nonlinearity for right and bilateral CPV supports use of the linear specification, but the proportional-hazards diagnostics suggest that their associations may be stronger earlier in follow-up. Moreover, calibration slopes below 1 and the absence of clear improvements in the bootstrap-validated C-index or time-dependent AUC indicates overfitting and no established incremental predictive value. The S2 analysis was restricted to 62 participants with both pre-index CSF biomarkers available. Because of the small sample, substantial biomarker missingness in the broader cohort, and the decision not to impute the CSF biomarkers, the S2 findings should be interpreted as exploratory. CPV should therefore not be interpreted as a stand-alone clinical prediction marker. The stage-specific pattern may indicate that CP abnormalities become more relevant after symptomatic disease is established, but it may also reflect accumulated disease severity. This interpretation is compatible with evidence that the choroid plexus participates in AD pathophysiology and that CP alterations may accompany impaired brain homeostasis [44]. Studies in an AD mouse model and human CP transcriptomic and CSF-protein datasets have reported CP cellular abnormalities, altered homeostatic gene expression, and associations between CPV and CSF protein levels [26,47,48]. These findings support altered CP homeostasis and tissue remodeling as candidate contexts for MRI-detectable CP enlargement, although the present observational analyses cannot establish the underlying tissue process.

To provide independent tissue-level context for the imaging findings, we analyzed single-nucleus and spatial transcriptomic datasets of the human choroid plexus from a postmortem cohort independent of ADNI. The module-ablation analysis showed that both epithelial states remained distinguishable after the curated genes were excluded before feature selection and clustering, indicating that their separation was not solely imposed by the predefined module. However, nucleus-level recoverability did not establish donor-level reproducibility: OCM-Epi-A was concentrated in three donors, whereas OCM-Epi-B was predominantly contributed by YA.1. Donor-level pseudobulk analyses that accounted for donor effects did not support reproducible pathway-level functional distinctions between the two states: no tested pathway met FDR < 0.05, and OCM-Epi-B had no qualifying protein-coding marker set for enrichment under the prespecified criteria. We therefore did not assign extracellular-matrix or proteostasis functions to OCM-Epi-A or OCM-Epi-B. Previous studies have linked elevated HCY to oxidative stress [38] and disruption of endothelial endoplasmic-reticulum redox homeostasis [39], but the present donor-level results did not establish corresponding state-specific pathway enrichment, and the absence of donor-specific HCY measurements precludes interpreting either state as an adaptive or downstream response to HCY. The narrow range of IRIS mixing proportions across four spatial libraries provided descriptive consistency under the restricted reference specification, but these libraries overlapped the single-nucleus donor cohort and the estimates remained reference-dependent. These states, particularly OCM-Epi-B, should therefore be interpreted as donor-sensitive, hypothesis-generating expression patterns requiring validation in larger independent cohorts. Spatial transcriptomic analyses also characterized the neighborhood organization of these expression states. OCM-Epi-A–like spots showed cross-sample neighborhood enrichment with endothelial spots, whereas the positive macrophage/myeloid association was observed in only one evaluable sample and was considered exploratory. Emerging evidence has established the CP as a important neuroimmune interface that regulates communication between peripheral inflammatory signals and the central nervous system [49]. In this context, the endothelial neighborhood enrichment may reflect local vascular–epithelial organization, although it does not establish HCY-related inflammation or coordinated cellular responses. Endothelial cells are major regulators of extracellular matrix (ECM) homeostasis and can secrete structural components such as collagens, laminins, and fibronectin, thereby influencing tissue architecture and mechanical stability [50]. Their spatial association with OCM-Epi-A–like spots is therefore consistent with a candidate vascular and ECM-related microenvironment but does not establish a role in CP expansion.

Ligand–receptor analysis further identified COL9A1–SDC4 as a higher-scoring predicted interaction between OCM-Epi-B and OCM-Epi-A. Both molecules have been implicated in extracellular matrix organization, mechanotransduction, focal adhesion formation, and tissue remodeling [51,52,53]. This analysis identifies COL9A1–SDC4 as a candidate expression-based ECM-related ligand–receptor pair between the two epithelial states. Taken together, the spatial neighborhood and ligand–receptor analyses provide hypothesis-generating context for the local microenvironment of these epithelial expression states. The endothelial neighborhood pattern and the candidate COL9A1–SDC4 pair should be interpreted as computational observations rather than evidence of coordinated signaling or tissue remodeling. Because the transcriptomic donors lacked documented HCY concentrations and individual diagnostic linkage, these analyses cannot establish HCY-induced metabolic shifts, validate the ADNI imaging associations, or define a molecular mechanism for CP enlargement.

This study has several strengths. First, by leveraging the ADNI cohort, we integrated clinical data, index and longitudinal structural MRI, and prospective diagnostic follow-up, enabling evaluation of cross-sectional HCY–CPV associations, index-CPV prognostic associations, and within-person CPV trajectories. In addition, the use of a systematic structural MRI phenotype-screening approach minimized the selection bias inherent in conventional candidate-region analyses. Second, beyond a single baseline measurement, we incorporated longitudinal HCY exposure metrics, including mean HCY levels and AUC, thereby providing a broader assessment of cumulative HCY exposure. Finally, reanalysis of independent single-nucleus and spatial transcriptomic datasets identified candidate epithelial expression states and their spatial context, providing biological context for hypotheses arising from the ADNI imaging findings.

Several limitations should also be acknowledged. First, the observational nature of this study precludes causal inference. Although repeated MRI measurements allowed us to evaluate pre-conversion CPV trajectories, the mixed-effects models used baseline rather than time-varying HCY and did not jointly model potentially informative truncation at diagnostic conversion. These analyses therefore do not establish whether HCY drives progressive CP enlargement or whether CPV temporally mediates clinical progression. Future studies should align repeated measurements of HCY, CPV, cognition, amyloid, phosphorylated tau, neurodegeneration, and treatment-related imaging changes and evaluate their temporal relationships using joint longitudinal–survival models. Longitudinal trajectory and change-point analyses may further clarify when CPV changes emerge relative to established AD biomarkers. A preliminary study of longitudinal barrier-focused MRI during anti-amyloid therapy illustrates one approach for monitoring treatment-associated imaging changes over time [54]. Second, missingness in BMI and laboratory covariates reduced the strict common complete-case samples, and differences between included and excluded participants indicate potential complete-case selection. Multiple imputation reduced this loss of information but relied on the missing-at-random assumption. Third, proportional-hazards violations in several MCI-to-AD models mean that the reported hazard ratios summarize average rather than constant associations over follow-up. Fourth, calibration slopes below 1 and reduced bootstrap stability in the higher-dimensional models indicate overfitting, and the prediction models have not undergone external validation. Finally, although the transcriptomic cohort was independent of ADNI, the spatial libraries originated from the same parent study and overlapped the single-nucleus donors. Similar IRIS estimates across these libraries therefore did not constitute independent donor replication or distinguish biological heterogeneity from sampling, regional, or reference-model effects. Future studies with linked donor-level HCY, folate, CPV, diagnostic, CP barrier function, and tissue-level measurements will be important for establishing the reproducibility, cross-modal correspondence, disease-stage specificity, and functional relevance of these epithelial states.

## 5. Conclusions

In conclusion, circulating HCY was associated with larger CPV, and larger CPV showed model-dependent associations with MCI-to-AD dementia progression in ADNI. Independent transcriptomic reanalysis identified one-carbon metabolism-enriched epithelial expression states and their spatial organization in postmortem CP tissue, providing hypothesis-generating tissue-level context for the ADNI associations. Studies with linked metabolic, imaging, diagnostic, and tissue-level measurements are warranted to evaluate the correspondence and functional significance of these observations.

## Figures and Tables

**Table 1:** Covariates included in the primary and sensitivity models.

Model Name	Covariates Included
C1 standard clinical model	Age at HCY index date; sex; years of education; apolipoprotein E (APOE) ε4 carrier status; body mass index; systolic blood pressure; diastolic blood pressure
C2 vascular/comorbidity model	C1 + hypertension history; hypertensive blood pressure flag; composite hypertension flag; diabetes history; smoking history; alcohol use history; cardiovascular disease history; stroke or transient ischemic attack history
C3 laboratory-adjusted model	C2 + creatinine; blood urea nitrogen; vitamin B12; glucose; triglycerides; total cholesterol; albumin; total protein; hemoglobin; hematocrit; thyroid-stimulating hormone
C4 medication-adjusted model	C3 + antihypertensive medication; B-vitamin or folate supplement; statin; antidiabetic medication; antiplatelet or anticoagulant medication
C1-N CPV neuroimaging sensitivity model	C1 + scanner batch; MRI field strength; laterality-matched lateral ventricular volume; total cerebrospinal fluid volume; total gray matter volume; laterality-matched hippocampal volume
S1 disease-severity sensitivity model	C1 + baseline Clinical Dementia Rating–Sum of Boxes (CDR-SB); scanner batch; laterality-matched hippocampal and entorhinal measures; white matter hypointensity burden; an alternative specification replaced CDR-SB with the Mini-Mental State Examination (MMSE).
S2 exploratory molecular-biomarker sensitivity model	S1 (CDR-SB specification) + observed pre-index cerebrospinal fluid amyloid-beta 42; observed pre-index cerebrospinal fluid phosphorylated tau. Restricted to participants with both biomarkers available.

## Data Availability

The clinical, neuroimaging, and biomarker data used in this study were obtained from the Alzheimer’s Disease Neuroimaging Initiative (ADNI) database (adni.loni.usc.edu). These data are available to qualified researchers upon application to the ADNI data access committee. The single-nucleus RNA sequencing and spatial transcriptomic datasets reanalyzed in this study are publicly available in the National Center for Biotechnology Information (NCBI) Gene Expression Omnibus (GEO) under the accession numbers GSE315551 and GSE315553 (BioProject PRJNA1398245). Processed data and customized analytical codes supporting the findings of this study are available from the corresponding author upon reasonable request.

## Supplementary Materials

The following supporting information can be downloaded at: https://www.mdpi.com/article/10.3390/biology15161423/s1, Supplementary Figures S1–S5; Supplementary Tables S1–S8. **Figure S1. Incremental prediction and observed-biomarker S2 analyses.** (A) Changes in Harrell’s C-index and inverse-probability-of-censoring-weighted 3- and 5-year time-dependent area under the curve after adding left, right, or bilateral choroid plexus volume (CPV) to the S1 model. (B) Hazard ratios per 1-standard-deviation higher CPV from the observed-biomarker S2 Cox models. (C) Changes in the C-index and 3- and 5-year time-dependent area under the curve after adding CPV to S2. Blue, orange, and green symbols denote left, right, and bilateral CPV, respectively. Circles show point estimates, and horizontal bars show 95% confidence or bootstrap percentile intervals. Gray dashed vertical lines indicate no change in panels A and C and a hazard ratio of 1 in panel B. Panels A and C are based on 500 participant-level bootstrap replicates. **Figure S2. Three- and five-year calibration of the CPV Cox models.** (A) CN-to-MCI cohort. (B) MCI-to-AD dementia cohort. Columns represent left, right, and bilateral CPV, and rows represent the C1, C2, C3, C4, and C1-N models. The horizontal axis shows mean predicted risk, and the vertical axis shows Kaplan–Meier observed risk. Blue circles and lines denote 3-year estimates, whereas brown squares and lines denote 5-year estimates. Vertical error bars show 95% confidence intervals for the Kaplan–Meier estimates. The gray dashed diagonal denotes perfect calibration. **Figure S3. Restricted cubic-spline assessment of CPV and clinical progression.** (A) CN-to-MCI cohort. (B) MCI-to-AD dementia cohort. Columns represent left, right, and bilateral intracranial-volume-residualized CPV, and rows represent the C1, C2, C3, C4, and C1-N models. The horizontal axis shows the CPV residual z-score, and the vertical axis shows the hazard ratio. Dark-blue curves show pooled hazard-ratio estimates, and light-blue shaded regions show 95% confidence intervals. Gray dashed horizontal lines indicate a hazard ratio of 1, and gray dotted vertical lines indicate the reference CPV residual z-score of 0. The displayed P value tests the nonlinear spline component. **Figure S4. Adjusted longitudinal trajectories of intracranial-volume-residualized CPV.** Columns represent left, right, and bilateral CPV, and rows represent the CN-to-MCI and MCI-to-AD dementia cohorts. The horizontal axis shows years since the index MRI, and the vertical axis shows adjusted intracranial-volume-residualized CPV in standard-deviation units. (A) Blue, gray, and orange lines denote baseline log-transformed homocysteine values at −1 standard deviation, the mean, and +1 standard deviation, respectively. (B) Blue and orange lines denote non-converters and converters, respectively. Shaded regions show 95% confidence intervals. Gray dashed horizontal lines indicate a CPV residual z-score of 0. **Figure S5. Module-exclusion reclustering, donor representation, and spatial projection of CP epithelial states.** (A) Workflow showing the original clustering and post hoc annotation procedures, removal of the curated 123-gene module, and the subsequent highly variable gene selection, dimensionality reduction, integration, and clustering steps. (B) Uniform Manifold Approximation and Projection of the module-excluded analysis. Teal and salmon points denote clusters C2 and C12, respectively; other nuclei are shown in gray. (C) Cross-tabulation of the original epithelial-state labels and module-independent clusters. Cell annotations provide nucleus counts and within-state percentages; darker blue indicates a larger within-state proportion. (D) Recovery metrics across combinations of random seeds and Leiden resolutions. Rows denote seed–resolution combinations, and columns show the adjusted Rand index, normalized mutual information, and state-specific F1 scores. Darker blue indicates a larger metric value. (E) Donor-level representation of OCM-Epi-A and OCM-Epi-B. Bars show the percentage of each donor’s frozen CP epithelial nuclei assigned to the corresponding state; labels above the bars give nucleus counts and percentages. (F) Mean IRIS-estimated mixing proportions of OCM-Epi-A and OCM-Epi-B across the four spatial samples. Teal and salmon bars denote OCM-Epi-A and OCM-Epi-B, respectively, and labels above the bars give the corresponding percentages. **Table S1. Participant-level temporal alignment of HCY measurements, index MRI, diagnostic conversion, and follow-up.** Columns report the publication-specific study identifier, diagnosis at the index MRI, index HCY-to-MRI interval, numbers of subsequent HCY measurements before, on, and after the MRI date, number of HCY measurements on or after conversion, conversion status and type, MRI-to-conversion interval, and follow-up duration. The index MRI date is defined as day 0; positive HCY-to-MRI intervals indicate that HCY preceded MRI. **Table S2. Associations between HCY and structural MRI phenotypes across the C1–C4 models.** Separate worksheets present the C1, C2, C3, and C4 models. Columns report MRI domain, MRI phenotype, hemisphere, sample size, standardized regression coefficient, 95% confidence interval, nominal P value, C1 global Benjamini–Hochberg false discovery rate, and intracranial-volume adjustment status. **Table S3. Associations of repeated pre-MRI HCY measures with structural MRI phenotypes.** Rows are organized by HCY exposure definition, covariate model, MRI category, and MRI phenotype. Columns report the standardized regression coefficient with its 95% confidence interval, nominal P value, Benjamini–Hochberg false discovery rate across 42 phenotypes, and Benjamini–Hochberg false discovery rate across 126 tests. The Definitions worksheet describes the analysis sample, exposure definitions, outcome scale, model structure, estimator, and multiple-testing families. **Table S4. Covariate missingness, complete-case selection, and strict common complete-case Cox analyses.** (A) Covariate-specific missingness, including candidate, available, and missing sample sizes and missing percentages. (B) Comparisons between included and excluded participants, including variable type, group-specific sample sizes and summaries, and standardized differences. (C) Strict common complete-case Cox-model results, including outcome, CPV laterality, model, participant and event counts, hazard ratio, 95% confidence interval, nominal P value, Benjamini–Hochberg false discovery rate, and testing-family size. The Notes worksheet defines the analysis population, missing-data handling, and false discovery rate correction. **Table S5. Observed-biomarker S2 Cox analysis.** Rows represent left, right, and bilateral CPV. Columns report the hazard ratio per 1-standard-deviation higher CPV, 95% confidence interval, nominal P value, and Benjamini–Hochberg false discovery rate. Notes specify the cerebrospinal fluid biomarker eligibility criteria and covariates included in the S2 model. **Table S6. Nonlinearity, calibration, and model diagnostics for the CPV Cox models.** (A) Restricted cubic-spline analyses, with columns for outcome, model, CPV measure, participant and event counts, knot locations, linear hazard ratio, linear P value, and P value for nonlinearity. (B) Calibration analyses, with columns for outcome, model, CPV measure, prediction horizon, mean predicted risk, Kaplan–Meier observed risk, predicted–observed difference, Brier score, calibration slope, and number of successful bootstrap attempts. (C) Proportional-hazards and influence diagnostics, including Schoenfeld-residual P values, numbers of proportional-hazards violations, maximum absolute DFBETA, influence threshold, convergence status, and model rank. The Notes worksheet provides definitions of the diagnostic measures. **Table S7. Longitudinal analyses of intracranial-volume-residualized CPV.** (A) Primary longitudinal analysis in the fixed outcome-specific cohorts. (B) Sensitivity analysis restricted to participants with at least two eligible MRI examinations. (C) Sensitivity analysis restricted to participants with observed temporally eligible baseline HCY. Columns in panels A–C report cohort, CPV measure, modeled effect, participant and event counts, number of MRI observations, estimate with 95% confidence interval, nominal P value, Benjamini–Hochberg false discovery rate, and random-effects structure. (D) Participant and serial-MRI observation flow. (E) Variable-specific missingness and imputation. (F) Linear mixed-effects model convergence and singularity diagnostics. The Definitions worksheet specifies the cohorts, MRI time window, outcomes, time scale, HCY exposure, and covariates. **Table S8. Donor-level transcriptomic analyses of OCM-Epi-A and OCM-Epi-B.** The Summary worksheet reports gene-filtering and marker-selection fields for each epithelial state. The Markers worksheet reports state, gene symbol, log_2_ fold change, nominal P value, gene-level Benjamini–Hochberg false discovery rate, target and reference detection proportions, and curated-module membership. The Nominal Pathways and All Pathways worksheets report gene-set library, pathway name, background and input gene counts, overlap statistics, nominal P value, and pathway-level false discovery rate. The leave-one-donor-out worksheet reports the removed donor, remaining nucleus count, adjusted Rand index, normalized mutual information, and cluster counts. The Definitions worksheet describes the comparison, independent analysis unit, statistical model, marker criteria, and enrichment settings.

## Abbreviations

AD	Alzheimer’s disease
ADNI	Alzheimer’s Disease Neuroimaging Initiative
ADSP	Alzheimer’s Disease Sequencing Project
APOE	Apolipoprotein E
ARI	Adjusted Rand index
AUC	Area under the curve
BH	Benjamini–Hochberg
Bilat	Bilateral
BMI	Body mass index
C1-N	CPV-specific neuroimaging sensitivity model
C1–C4	Sequential covariate-adjustment models defined in Table 1
CDR-SB	Clinical Dementia Rating–Sum of Boxes
CI	Confidence interval
CN	Cognitively normal
CP	Choroid plexus
CPV	Choroid plexus volume
CSF	Cerebrospinal fluid
DFBETA	Change in a regression coefficient after deletion of one observation
ECM	Extracellular matrix
ENIGMA	Enhancing Neuroimaging Genetics through Meta-Analysis
F1	Harmonic mean of precision and recall
FDR	False discovery rate
GEO	Gene Expression Omnibus
GO	Gene Ontology
HC3	Type 3 heteroscedasticity-consistent covariance estimator
HCY	Homocysteine
HR	Hazard ratio
HVG	Highly variable gene
ICV	Intracranial volume
IR-SPGR	Inversion recovery–prepared spoiled gradient-recalled echo
IRIS	Integrative and reference-informed tissue segmentation
KEGG	Kyoto Encyclopedia of Genes and Genomes
L	Left
LIANA	Ligand–receptor analysis framework
MCI	Mild cognitive impairment
MMSE	Mini-Mental State Examination
MPRAGE	Magnetization-prepared rapid gradient-echo
MRI	Magnetic resonance imaging
MT	Middle temporal
NCBI	National Center for Biotechnology Information
NMI	Normalized mutual information
ns	Not significant
OCM	One-carbon metabolism
OCM-Epi-A	One-carbon metabolism-enriched epithelial state A
OCM-Epi-B	One-carbon metabolism-enriched epithelial state B
PCA	Principal component analysis
PHC	Phenotype Harmonization Consortium
QC	Quality control
R	Right
RRR	Relative risk ratio
S1	Disease-severity sensitivity model
S2	Molecular-biomarker sensitivity model
SD	Standard deviation
SMC	Smooth muscle cell
snRNA-seq	Single-nucleus RNA sequencing
SRA	Sequence Read Archive
TMMwsp	Trimmed mean of M values with singleton pairing
TSH	Thyroid-stimulating hormone
UMAP	Uniform Manifold Approximation and Projection
