# Supplementary material for: Circulating Homocysteine and Choroid Plexus Volume Across the Alzheimer’s Disease Continuum: Cross-Sectional and Progression-Related Associations
Source: Biology (Basel). 2026 Aug 18;15(16):1423. doi: 10.3390/biology15161423 (PMC13509176; doi:10.3390/biology15161423)
Supplement: Supplementary file 1 [file biology-15-01423-s001.zip › Supplementary_Figure_S1_CPV_Progression_Sensitivity.pdf]

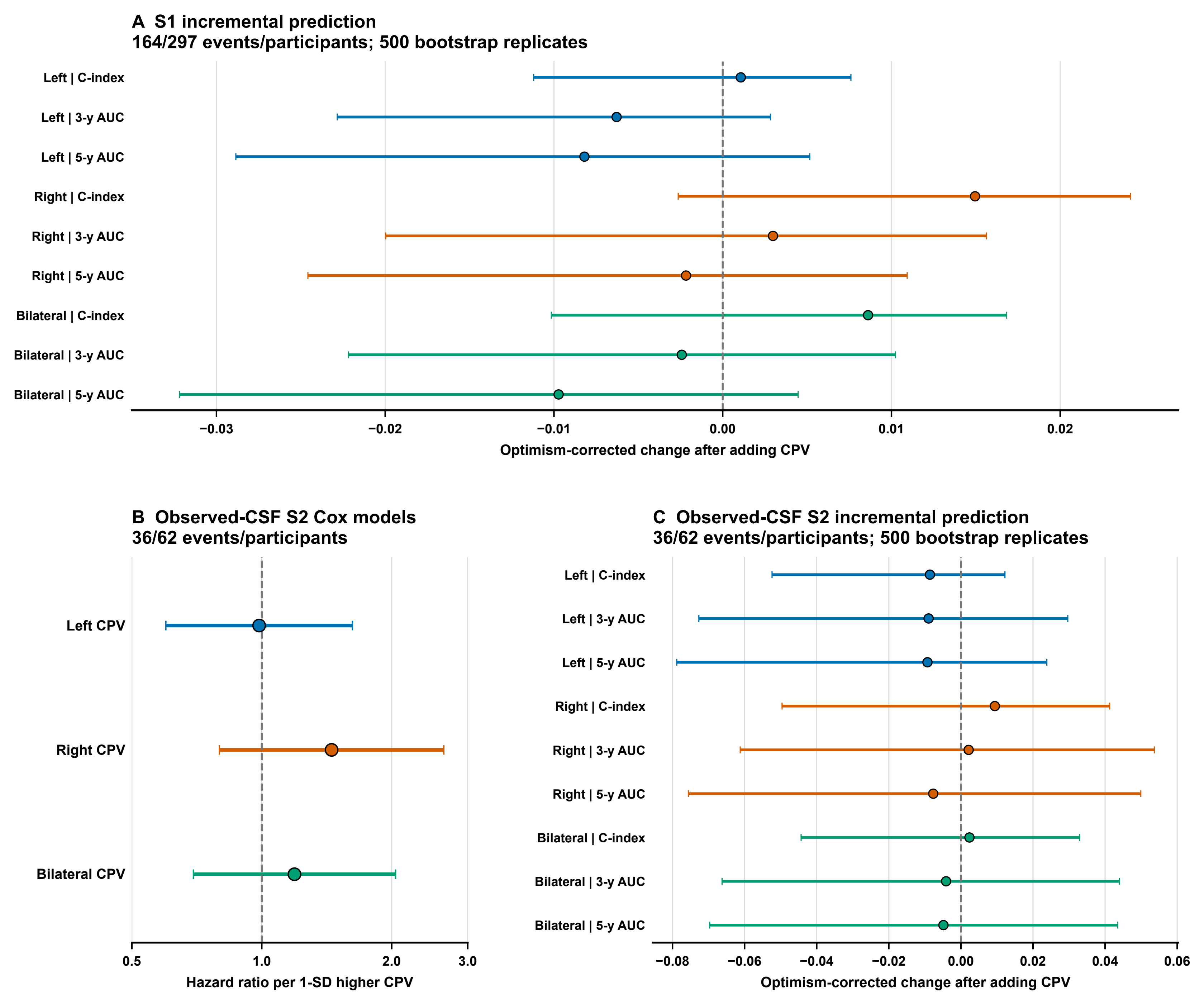

A S1 incremental prediction
164/297 events/participants; 500 bootstrap replicates
Left | C-index
Left | 3-y AUC
Left | 5-y AUC
Right | C-index
Right | 3-y AUC
Right | 5-y AUC
Bilateral | C-index
Bilateral | 3-y AUC
Bilateral | 5-y AUC
−0.03
−0.02
−0.01
0.00
0.01
0.02
Optimism-corrected change after adding CPV
B Observed-CSF S2 Cox models
36/62 events/participants
Left CPV
Right CPV
Bilateral CPV
0.5
1.0
2.0
3.0
Hazard ratio per 1-SD higher CPV
C Observed-CSF S2 incremental prediction
36/62 events/participants; 500 bootstrap replicates
Left | C-index
Left | 3-y AUC
Left | 5-y AUC
Right | C-index
Right | 3-y AUC
Right | 5-y AUC
Bilateral | C-index
Bilateral | 3-y AUC
Bilateral | 5-y AUC
−0.08
−0.06
−0.04
−0.02
0.00
0.02
0.04
0.06
Optimism-corrected change after adding CPV
