# Supplementary material for: Circulating Homocysteine and Choroid Plexus Volume Across the Alzheimer’s Disease Continuum: Cross-Sectional and Progression-Related Associations
Source: Biology (Basel). 2026 Aug 18;15(16):1423. doi: 10.3390/biology15161423 (PMC13509176; doi:10.3390/biology15161423)

A CN to MCI

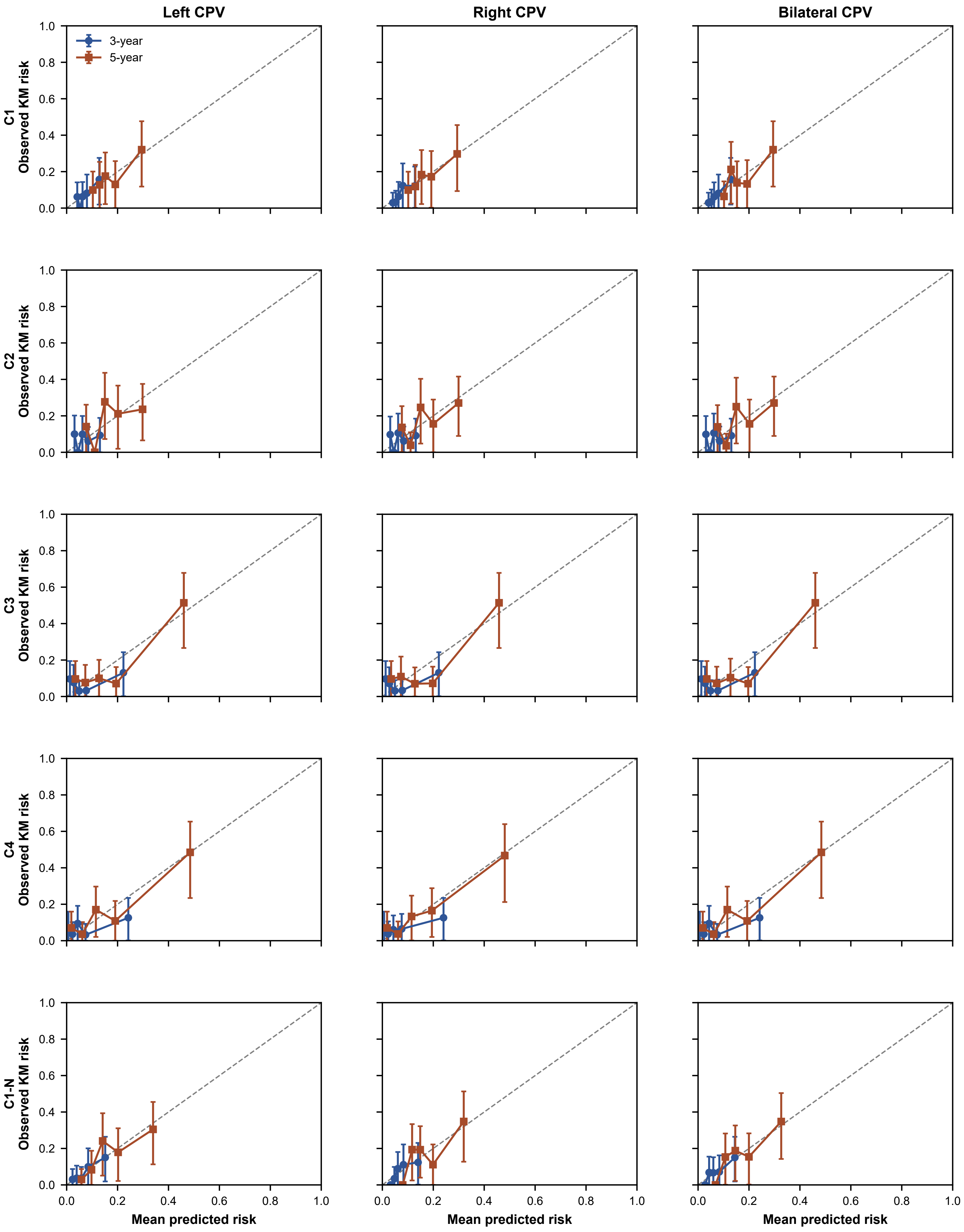

Left CPV
Right CPV
Bilateral CPV
3-year
5-year
C1
C2
C3
C4
C1-N
Observed KM risk
Mean predicted risk
0.0
0.2
0.4
0.6
0.8
1.0

B MCI to AD dementia

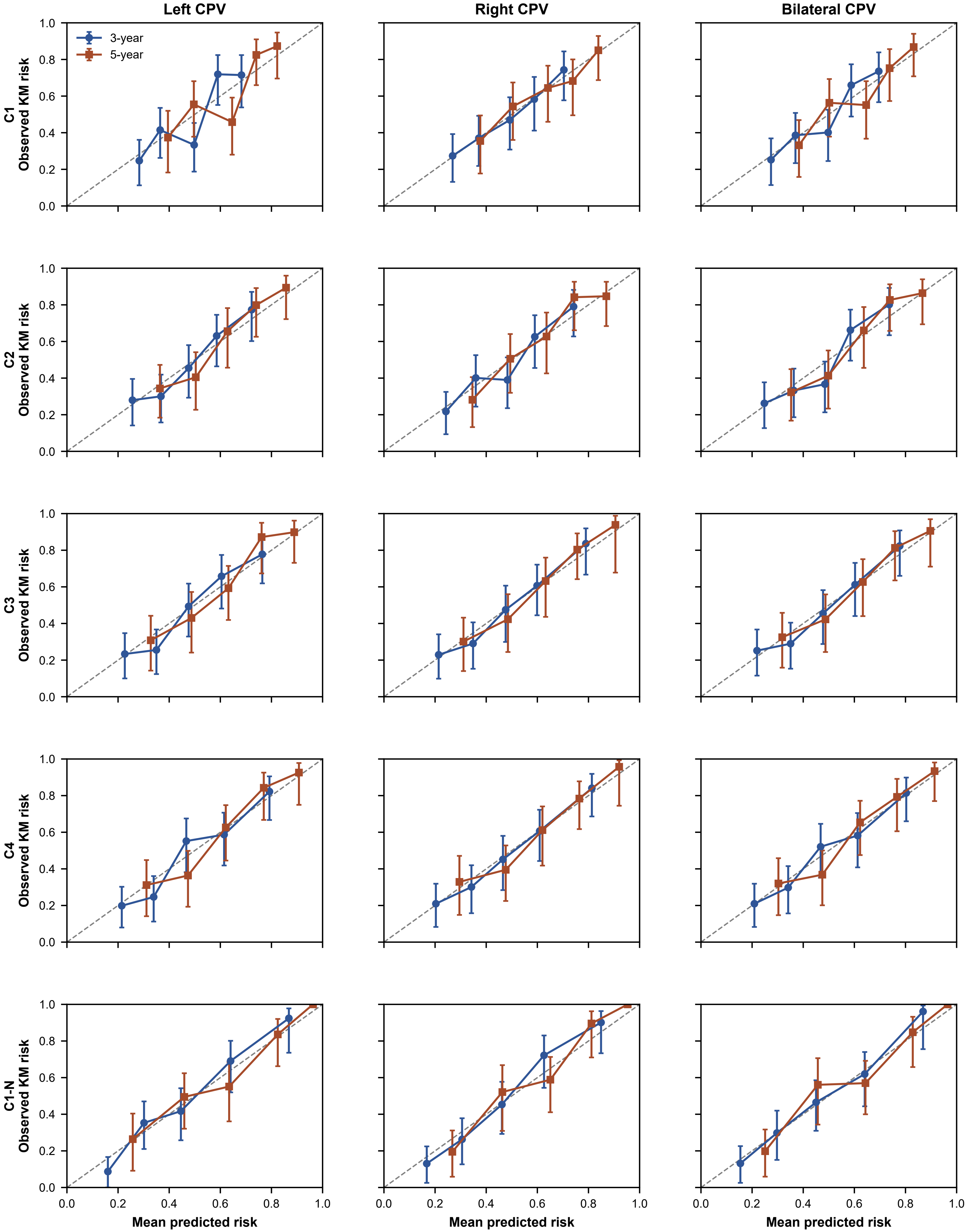

Supplement: Supplementary file 1 [file biology-15-01423-s001.zip › Supplementary_Figure_S2_Model_Calibration.pdf]
