# Supplementary material for: Circulating Homocysteine and Choroid Plexus Volume Across the Alzheimer’s Disease Continuum: Cross-Sectional and Progression-Related Associations
Source: Biology (Basel). 2026 Aug 18;15(16):1423. doi: 10.3390/biology15161423 (PMC13509176; doi:10.3390/biology15161423)
Supplement: Supplementary file 1 [file biology-15-01423-s001.zip › Supplementary_Figure_S3_CPV_Nonlinearity.pdf]

A CN to MCI

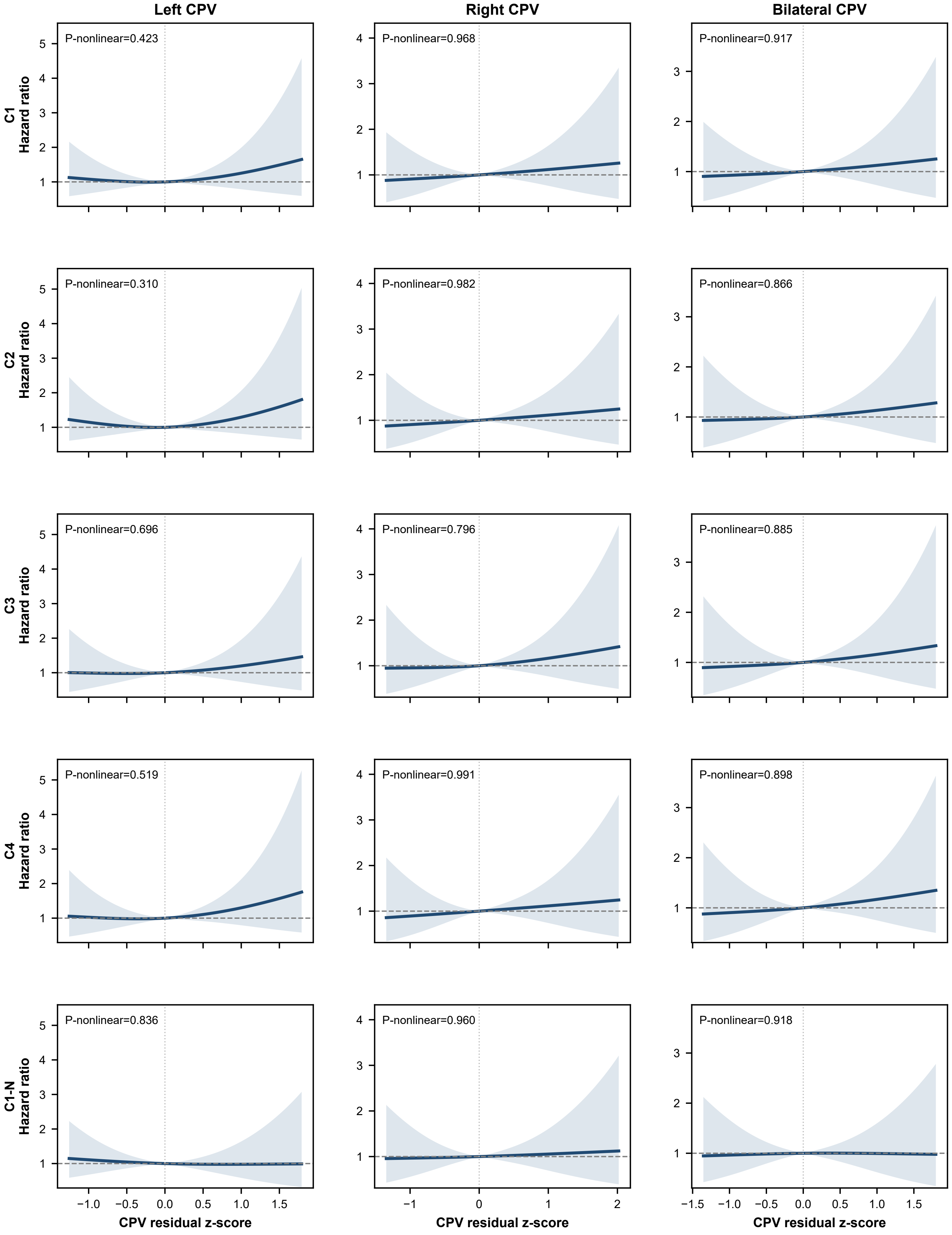

Left CPV
Right CPV
Bilateral CPV
C1 Hazard ratio
C2 Hazard ratio
C3 Hazard ratio
C4 Hazard ratio
C1-N Hazard ratio
P-nonlinear=0.423
P-nonlinear=0.968
P-nonlinear=0.917
P-nonlinear=0.310
P-nonlinear=0.982
P-nonlinear=0.866
P-nonlinear=0.696
P-nonlinear=0.796
P-nonlinear=0.885
P-nonlinear=0.519
P-nonlinear=0.991
P-nonlinear=0.898
P-nonlinear=0.836
P-nonlinear=0.960
P-nonlinear=0.918
CPV residual z-score
CPV residual z-score
CPV residual z-score

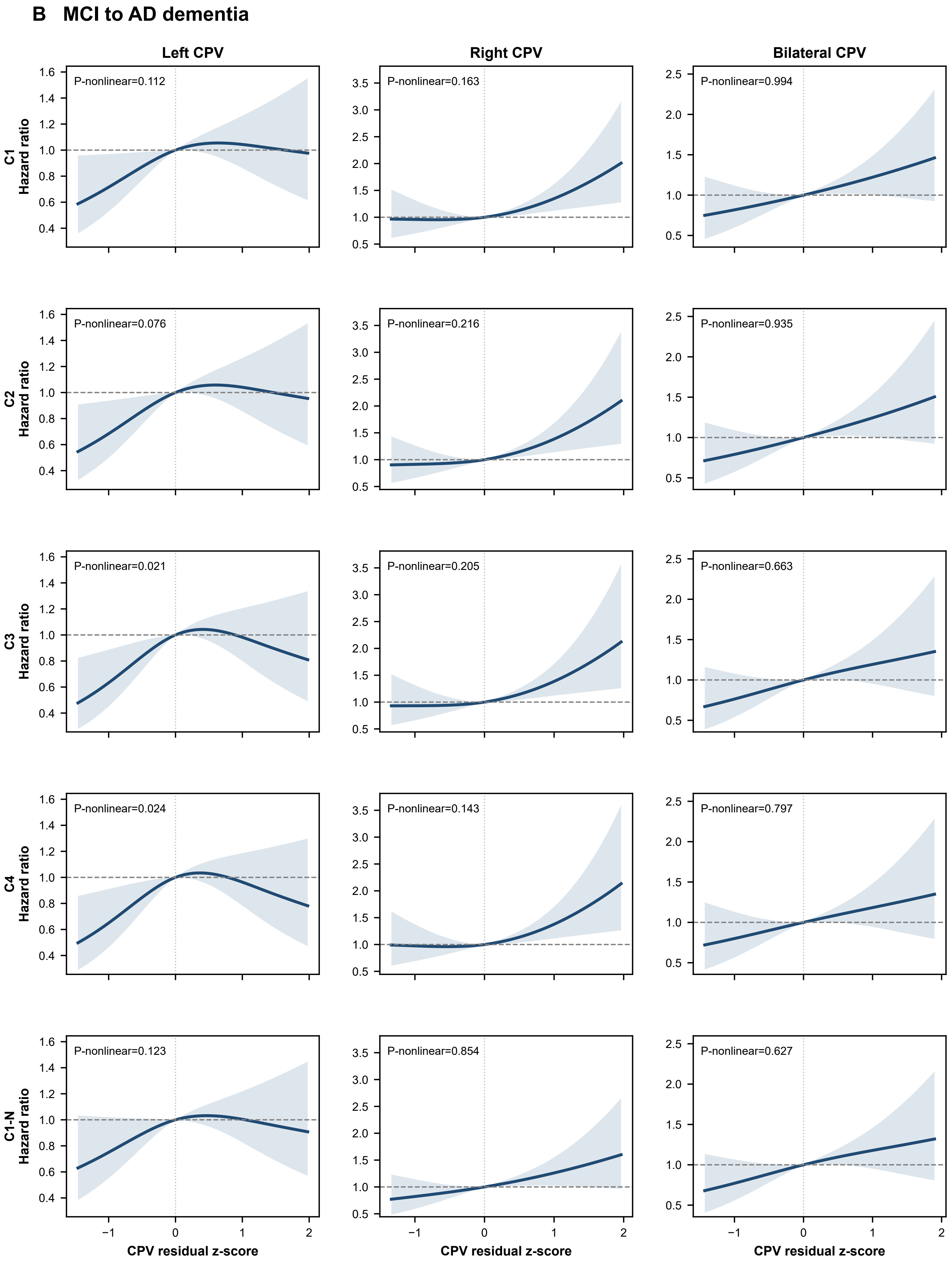
B MCI to AD dementia
Left CPV
Right CPV
Bilateral CPV
C1
C2
C3
C4
C1-N
Hazard ratio
P-nonlinear=0.112
P-nonlinear=0.163
P-nonlinear=0.994
P-nonlinear=0.076
P-nonlinear=0.216
P-nonlinear=0.935
P-nonlinear=0.021
P-nonlinear=0.205
P-nonlinear=0.663
P-nonlinear=0.024
P-nonlinear=0.143
P-nonlinear=0.797
P-nonlinear=0.123
P-nonlinear=0.854
P-nonlinear=0.627
CPV residual z-score
