# Supplementary material for: Circulating Homocysteine and Choroid Plexus Volume Across the Alzheimer’s Disease Continuum: Cross-Sectional and Progression-Related Associations
Source: Biology (Basel). 2026 Aug 18;15(16):1423. doi: 10.3390/biology15161423 (PMC13509176; doi:10.3390/biology15161423)
Supplement: Supplementary file 1 [file biology-15-01423-s001.zip › Supplementary_Figure_S4_Longitudinal_CPV_Trajectories.pdf]

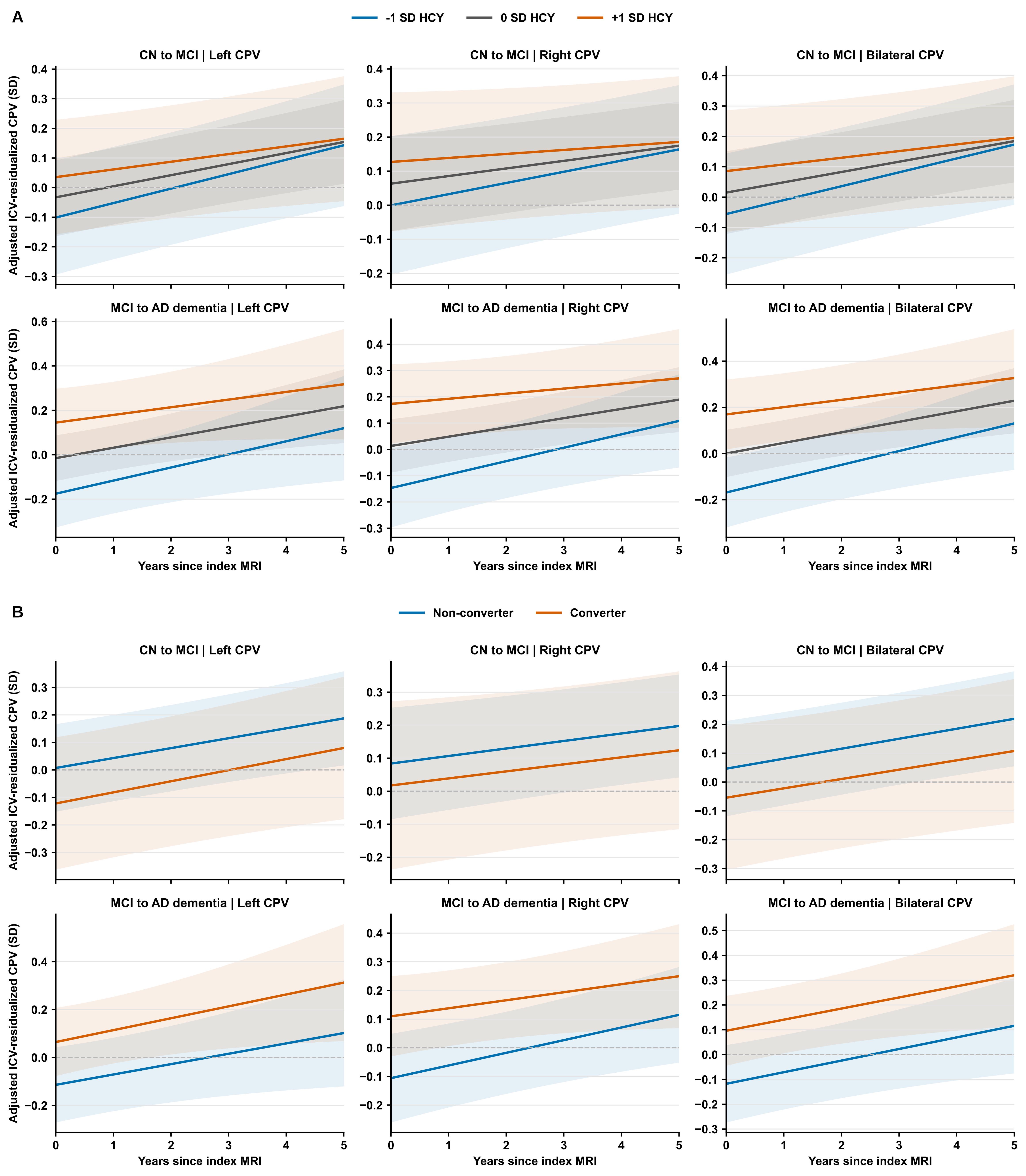

A
-1 SD HCY
0 SD HCY
+1 SD HCY
CN to MCI | Left CPV
CN to MCI | Right CPV
CN to MCI | Bilateral CPV
MCI to AD dementia | Left CPV
MCI to AD dementia | Right CPV
MCI to AD dementia | Bilateral CPV
Adjusted ICV-residualized CPV (SD)
Years since index MRI
B
Non-converter
Converter
CN to MCI | Left CPV
CN to MCI | Right CPV
CN to MCI | Bilateral CPV
MCI to AD dementia | Left CPV
MCI to AD dementia | Right CPV
MCI to AD dementia | Bilateral CPV
Adjusted ICV-residualized CPV (SD)
Years since index MRI
