# Supplementary material for: Circulating Homocysteine and Choroid Plexus Volume Across the Alzheimer’s Disease Continuum: Cross-Sectional and Progression-Related Associations
Source: Biology (Basel). 2026 Aug 18;15(16):1423. doi: 10.3390/biology15161423 (PMC13509176; doi:10.3390/biology15161423)
Supplement: Supplementary file 1 [file biology-15-01423-s001.zip › Supplementary_Figure_S5_Module_Independent_Recovery_Donor_Representation.pdf]

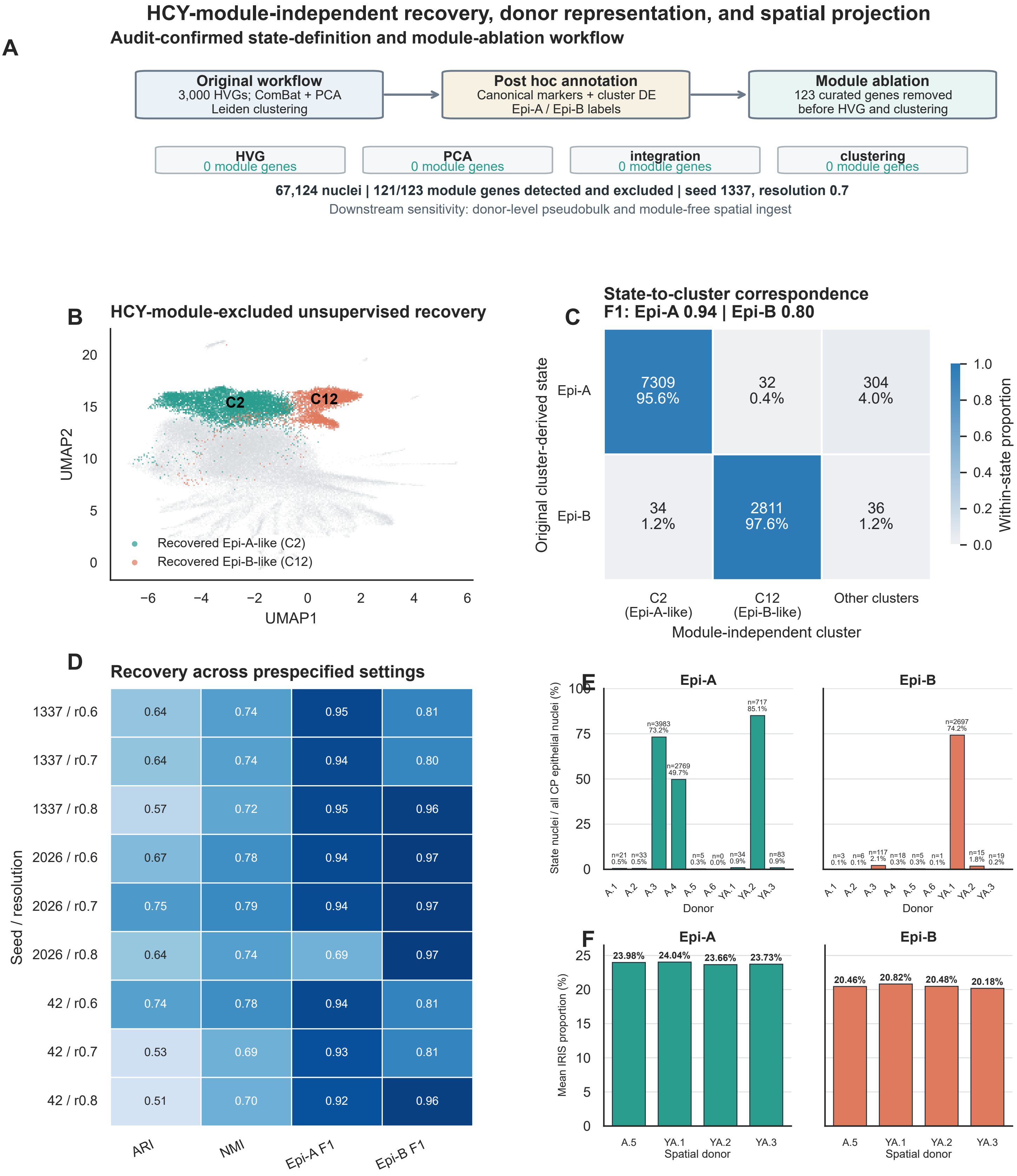

HCY-module-independent recovery, donor representation, and spatial projection
A
Audit-confirmed state-definition and module-ablation workflow
Original workflow
3,000 HVGs; ComBat + PCA
Leiden clustering
Post hoc annotation
Canonical markers + cluster DE
Epi-A / Epi-B labels
Module ablation
123 curated genes removed
before HVG and clustering
HVG
0 module genes
PCA
0 module genes
integration
0 module genes
clustering
0 module genes
67,124 nuclei | 121/123 module genes detected and excluded | seed 1337, resolution 0.7
Downstream sensitivity: donor-level pseudobulk and module-free spatial ingest
B
HCY-module-excluded unsupervised recovery
C2
C12
Recovered Epi-A-like (C2)
Recovered Epi-B-like (C12)
UMAP1
UMAP2
C
State-to-cluster correspondence
F1: Epi-A 0.94 | Epi-B 0.80
Original cluster-derived state
Epi-A
7309 95.6%
32 0.4%
304 4.0%
Epi-B
34 1.2%
2811 97.6%
36 1.2%
C2 (Epi-A-like)
C12 (Epi-B-like)
Other clusters
Module-independent cluster
Within-state proportion
D
Recovery across prespecified settings
Seed / resolution
ARI NMI Epi-A F1 Epi-B F1
1337 / r0.6 0.64 0.74 0.95 0.81
1337 / r0.7 0.64 0.74 0.94 0.80
1337 / r0.8 0.57 0.72 0.95 0.96
2026 / r0.6 0.67 0.78 0.94 0.97
2026 / r0.7 0.75 0.79 0.94 0.97
2026 / r0.8 0.64 0.74 0.69 0.97
42 / r0.6 0.74 0.78 0.94 0.81
42 / r0.7 0.53 0.69 0.93 0.81
42 / r0.8 0.51 0.70 0.92 0.96
E
Epi-A
n=21 0.5% | n=33 0.5% | n=3983 73.2% | n=2769 49.7% | n=5 0.3% | n=0 0.0% | n=34 0.9% | n=717 85.1% | n=83 0.9%
Epi-B
n=3 0.1% | n=6 0.1% | n=117 2.1% | n=18 0.3% | n=5 0.3% | n=1 0.1% | n=2697 74.2% | n=15 1.8% | n=19 0.2%
A.1 A.2 A.3 A.4 A.5 A.6 YA.1 YA.2 YA.3
Donor
State nuclei / all CP epithelial nuclei (%)
F
Epi-A
23.98% 24.04% 23.66% 23.73%
Epi-B
20.46% 20.82% 20.48% 20.18%
A.5 YA.1 YA.2 YA.3
Spatial donor
Mean IRIS proportion (%)
